# Comparative Ultrasonic Bath and Probe Extraction of Piperine from *Piper nigrum* L. Using Natural Deep Eutectic Solvents: RSM Optimization, Characterization, and In Vitro Bioactivity

**DOI:** 10.3390/biom15111631

**Published:** 2025-11-20

**Authors:** Abdullah Mohammed Ayedh Al Adhreai, Johnson Retnaraj Samuel Selvan Christyraj, Beryl Vedha Yesudhason, Yolin Angel Poomany Arul Soundara Rajan, Maharshi Bhaswant

**Affiliations:** 1 Regeneration and Stem Cell Biology Lab, Centre for Molecular and Nanomedical Sciences, International Research Centre, Sathyabama Institute of Science and Technology, Chennai 600119, Tamil Nadu, India; 2 Centre for Nanoscience and Nanotechnology, Sathyabama Institute of Science and Technology, Chennai 600119, Tamil Nadu, India; 3 School of Biosciences and Technology, SRM Institute of Science and Technology, Tiruchirappalli Campus, Tiruchirappalli 621105, Tamil Nadu, India

**Keywords:** piperine, NADES, ultrasonic bath, ultrasonic probe, RSM optimization, anticancer activity, antioxidant activity

## Abstract

Background: *Piper nigrum* L. (PNL) is a rich source of piperine, a bioactive alkaloid with pharmaceutical, cosmetic, nutritional supplement, and agricultural applications, yet efficient and sustainable extraction methods remain underexplored. Methods: This study compared ultrasonic bath extraction (UBE) and ultrasonic probe extraction (UPE) using natural deep eutectic solvents (NADES) for isolating piperine from PNL fruits. Six NADES formulations were screened, with NADES-5 (choline chloride:glycerin:urea, 1:1:1) showing superior performance. Response surface methodology with a Box–Behnken design optimized extraction parameters, including liquid-to-solid ratio, extraction time, temperature, and water content, for both UBE and UPE. Results: Optimized UPE consistently outperformed UBE, yielding 49.97 mg/g of piperine versus 25.67 mg/g under identical NADES conditions. Comprehensive characterization using TLC, HPTLC, UV, FTIR, Raman, HPLC, NMR, XRD, SEM, and EDX confirmed the successful isolation and structural integrity of piperine, with samples obtained via UPE exhibiting higher purity (98.7% vs. 95.2%) and enhanced crystallinity. In vitro cytotoxicity assays demonstrated that piperine extracted by UPE showed stronger activity against C2C12 myoblasts (IC_50_: 24.3 μg/mL vs. 40.6 μg/mL) and greater anticancer effects in MCF-7 and HT-29 cells compared to piperine extracted by UBE. Antioxidant evaluation via DPPH, ABTS, FRAP, and TAC assays, along with intracellular reactive oxygen and nitrogen species suppression in THP-1 and RAW 264.7 macrophages, further confirmed the superior biological potential of the UPE-derived piperine sample. Conclusions: These findings indicate that UPE using NADES is a sustainable approach for high-yield piperine extraction with enhanced purity and bioactivity, supporting its potential for pharmaceutical applications.

## 1. Introduction

PNL, also called black pepper, is one of the most widely used spices across the globe, and is valued not only for its culinary pungency but also for its medicinal and preservative properties. It belongs to the *Piperaceae* family and is rich in bioactive phytochemicals, the most important of which is piperine, a yellow crystalline alkaloid with the molecular formula C_17_H_19_NO_3_. Piperine is not exclusive to *Piper nigrum*; it also occurs in other *Piper* species such as *P. longum* (long pepper) and *P. retrofractum*. However, its concentration in these species is generally lower than that found in *P. nigrum*, which remains the most abundant natural source of piperine due to its higher alkaloid biosynthetic capacity and fruit yield. Piperine accounts for the sharp taste of pepper and has attracted considerable pharmacological interest due to its diverse therapeutic effects [1,2,3,4]. Beyond its biomedical importance, piperine also finds emerging applications in cosmetics, pest management, and agrochemical formulations owing to its natural bioactive properties [5,6,7]. Extensive research has demonstrated its antioxidant, anti-inflammatory, anticancer, antimicrobial, neuroprotective, immunomodulatory, and hepatoprotective properties, supporting its potential as a nutraceutical and pharmaceutical agent [8,9,10,11,12,13]. Despite these promising bioactivities, raw pepper contains only 3–9% piperine, and direct consumption is insufficient to achieve the therapeutic doses required for clinical applications [3,14]. Therefore, the development of efficient, scalable, and sustainable extraction strategies for piperine remains a critical area of research.

Traditional extraction methods such as Soxhlet extraction, maceration, and percolation have been widely applied for alkaloid recovery from medicinal plants, but these approaches suffer from major drawbacks, including high solvent consumption, long processing times, elevated temperatures that can degrade thermolabile compounds, and relatively low yields [15,16,17,18,19,20]. In recent years, ultrasound-assisted extraction (UAE) has emerged as a promising green technology that overcomes many of these limitations. The principle of UAE lies in acoustic cavitation, wherein ultrasonic waves generate microbubbles that collapse violently, producing localized high temperatures and pressures. This phenomenon disrupts plant cell walls, enhances solvent penetration, and accelerates the release of intracellular metabolites into the solvent [21].

Within UAE, two common modalities are employed: ultrasonic bath extraction (UBE) and ultrasonic probe extraction (UPE). UBE is widely used due to its simplicity, cost-effectiveness, and ability to provide uniform energy distribution, but its relatively low intensity can limit extraction efficiency [22]. In contrast, UPE delivers high-intensity, localized ultrasonic energy that promotes greater cell disruption and solvent diffusion, often resulting in superior yields within significantly shorter extraction times [19,23]. Recent studies have demonstrated that probe-based sonication enhances both the extraction efficiency and bioactivity of phytochemicals from spice plants. According to Zorrilla et al. (2024), sonication-assisted extraction markedly improved the extraction of phenolic and terpenoid constituents from *Zingiber officinale* (ginger), thereby augmenting its antioxidant and bioherbicidal potential [24]. Similarly, Shaterabadi et al. (2020) observed that ultrasound-assisted extraction using continuous and pulsed sonication modalities applied to black caraway (*Carum carvi* L.) seeds showed that higher ultrasound power intensities (100–300 W) and extended sonication durations (10–20 min) resulted in significantly increased dry extract yields and ascorbic acid content compared to conventional Soxhlet extraction, confirming the superiority of ultrasonic treatment for alkaloid and bioactive compound recovery from medicinal spices [25]. Additionally, Sganzerla et al. (2014) established that ultrasonic extraction provided an excellent method for quantitative and reproducible extraction of capsaicinoids from chili peppers (*Capsicum* species) using methanol as a solvent, achieving a full-spectrum extract with superior bioactive profiles and significantly higher yields in much shorter processing times than traditional heat-reflux methods [26]. Collectively, these investigations illustrate the substantial advantages of ultrasonic extraction modalities for the recovery of diverse alkaloids and phytochemicals from spice plants, leading to improved extraction efficiency and bioactive compound preservation. However, few studies have systematically compared the relative performance of UBE and UPE for piperine recovery under identical experimental conditions.

In parallel with advances in extraction technology, the development of environmentally friendly solvents has further accelerated the transition toward sustainable extraction systems. Natural deep eutectic solvents (NADES) have emerged as a novel class of green solvents, typically composed of hydrogen bond donors and acceptors such as choline chloride, urea, sugars, and organic acids. NADES are biodegradable, inexpensive, easy to prepare, and tunable in polarity, making them particularly effective for solubilizing diverse bioactive compounds [27,28]. Compared to conventional organic solvents, NADES exhibit reduced toxicity, improved thermal stability, and enhanced ability to preserve the structural integrity of labile molecules. Notably, Masala et al. (2024) demonstrated that ultrasound-assisted extraction using NADES substantially improved the recovery of phenolic compounds from saffron (*Crocus sativus*) floral by-products, leading to enhanced antioxidant capacity and extract quality [29]. Furthermore, Lwamba et al. (2023) [30] developed a novel NADES-based ultrasound-assisted extraction approach for piperine recovery from black pepper (*Piper nigrum* L.), employing a choline chloride-citric acid-1,2-propylene glycol combination (1:2:2 molar ratio) with optimized water content (25% *v*/*v*). This innovative approach achieved a piperine extraction yield of 39.075 mg/g—significantly superior to conventional Soxhlet extraction (39.1 mg/g) and ethanol extraction (46.6 mg/g)—while producing a high-purity extract (90%) with substantially enhanced antioxidant activity (45.34% DPPH inhibition) compared to water, ethanol, and methanol controls [30]. Similarly, Stasiłowicz-Krzemień et al. (2024) reported that combining NADES with supercritical CO_2_ extraction for turmeric (*Curcuma longa* L.) curcuminoid recovery yielded 33.35 mg/g of curcuminoids substantially outperforming conventional alcohol solvents (22.95–26.42 mg/g) and demonstrating the effectiveness of eutectic mixtures, particularly menthol–lactic acid formulations (1:2 molar ratio), in extracting thermolabile alkaloid and polyphenolic compounds while preserving their bioactivity [31]. Of the NADES formulations, choline chloride-based systems are most frequently employed due to their safety, affordability, and strong hydrogen-bonding capabilities.

Recent studies have validated the efficiency of NADES in extracting polyphenols, alkaloids, and flavonoids from complex plant matrices including spice residues and medicinal herbs, demonstrating their suitability for piperine extraction [32,33,34,35]. The superior extraction performance of NADES formulations has been attributed to their ability to form strong hydrogen bonds with target compounds, enhance mass transfer rates through their tunable viscosity, and stabilize bioactive molecules against thermal or oxidative degradation that typically occurs with conventional extraction methods.

To maximize extraction efficiency and ensure reproducibility, statistical optimization methods such as response surface methodology (RSM) have become indispensable tools. RSM enables the simultaneous evaluation of multiple experimental factors and their interactions, thereby reducing the number of required trials while improving the accuracy of predictive models [23,36]. Within RSM, the Box–Behnken design (BBD) is particularly advantageous for extraction studies, as it provides rotatability, requires fewer experimental runs than central composite designs, and allows estimation of quadratic effects [36,37,38]. By optimizing critical parameters such as liquid-to-solid ratio, extraction time, temperature, and water content in NADES, RSM can significantly enhance the yield and purity of target compounds [23,36,39]. While RSM has been widely applied to optimize extraction of polyphenols, flavonoids, and essential oils, its application to piperine extraction using NADES remains underexplored, especially when comparing UBE and UPE as complementary techniques.

Despite these technological advances, a major research gap persists in the integrated evaluation of extraction efficiency, solvent systems, and biological activity. Most previous studies have focused primarily on optimizing yield or solvent performance, without systematically assessing how the extraction methodology influences the purity, crystallinity, and pharmacological potential of extracted piperine. In particular, there is limited evidence comparing how UBE and UPE affect not only extraction yield but also the antioxidant and anticancer activities of purified piperine when green solvents are employed. Addressing this gap is essential for developing sustainable extraction protocols that maximize both chemical recovery and therapeutic efficacy.

Accordingly, the present study was designed with four major objectives: (i) to screen and identify the most effective NADES formulation for piperine extraction from PNL fruits collected in Chennai, India; (ii) to apply RSM-BBD for optimizing critical process parameters; (iii) to conduct a comparative evaluation of UBE and UPE in terms of piperine yield, purity, and crystallinity; and (iv) to assess the in vitro bioactivity of purified extracts, including antioxidant and anticancer activities, alongside comprehensive characterization using TLC, UV-Vis, FTIR, HPLC, HPTLC, Raman spectroscopy, NMR, SEM, EDX, and XRD. Based on the advantages of probe-assisted UAE and NADES, we hypothesize that UPE will yield piperine of higher purity with superior bioactivity compared to UBE. The novelty of this study lies in its bioactivity-driven optimization framework, its comparative assessment of ultrasonic modalities using identical NADES formulations, and its integration of chemical, structural, and pharmacological evaluations. To the best of our knowledge, this is the first systematic study to couple UPE and NADES with RSM for piperine isolation, offering new insights into sustainable extraction methodologies for pharmaceutical and nutraceutical applications.

## 2. Materials and Methods

### 2.1. Chemicals and Reagents

All chemicals and solvents used in this study were of analytical grade. Choline chloride, citric acid, malic acid, glycerin, 1,2-propylene glycol, urea, and L-proline (NADES components) were purchased from Sigma-Aldrich (Bangalore, India). Ethanol, dichloromethane, methanol, and other solvents were obtained from Sri Amman Scientific and Surgical (SASS, Chennai, India). Doxorubicin and IL-1β were also procured from Sigma-Aldrich (Bangalore, India). The C2C12 mouse myoblasts, RAW 264.7 murine macrophages, and THP-1 human monocytes were obtained from the National Centre for Cell Science (NCCS, Pune, India). Cell culture reagents, including Dulbecco’s Modified Eagle Medium (DMEM), RPMI-1640 medium, fetal bovine serum (FBS), penicillin–streptomycin, and trypsin-EDTA, as well as the MTT reagent, DCFH-DA probe, and BBoxiProbe™ R21F kit, were obtained from Sigma-Aldrich (Bangalore, India) and SASS (Chennai, India). Reagents used in antioxidant assays, including ABTS, DPPH, FeSO_4_, and phosphomolybdate, were of analytical grade. Ultrapure water (Milli-Q system, Millipore, Burlington, MA, USA) was used for all preparations.

### 2.2. Plant Material

Fruits of PNL, collected from Kanyakumari, Tamil Nadu, India, were authenticated by Dr. S. Rajan, Central Council for Research in Homoeopathy, Ministry of AYUSH, Government of India. The seeds were cleaned, air-dried, and pulverized into fine powder using an electric grinder. The powder was stored at 37 °C in a sealed container until further use for extraction and analysis.

### 2.3. Preparation of NADES

Six different NADES were selected for the extraction of piperine from PNL fruits. Their detailed compositions, including Hydrogen Bond Acceptors (HBA), Hydrogen Bond Donors (HBD), and their molar ratios, are listed in Table 1. Each NADES was prepared by accurately weighing the individual components and mixing them under constant magnetic stirring at 80 °C until a clear and homogeneous liquid was formed. After preparation, the mixtures were cooled to room temperature and stored in airtight containers. The stability of all NADES was confirmed by observing no phase separation or precipitation under ambient conditions for at least 24 h prior to use. For the extraction processes, a fixed sample-to-solvent ratio of 1:15 (*w*/*v*) was maintained. The same set of six NADES was employed for both UBE and UPE, selected based on their previously reported extraction efficiency, favorable physicochemical properties (such as low viscosity and stability), and compatibility with ultrasound-assisted extraction techniques [40,41].

### 2.4. Preliminary Extraction of Piperine by UBE and UPE

The preliminary extraction was performed to evaluate the efficiency of both UBE and UPE using NADES as green extraction media for piperine from PNL fruits [4,42]. Briefly, 1.0 g of pulverized PNL seed powder was mixed with 15 mL of a selected NADES at a fixed sample-to-solvent ratio of 1:15 (*w*/*v*). For UBE, extraction was carried out in an ultrasonic bath (Elmasonic S 60 H, Elma Schmidbauer GmbH, Singen, Germany) operating at 40 kHz frequency and 200 W power at 45 °C for 30 min. For UPE, extraction was conducted using an ultrasonic probe system (VCX 500, Sonics & Materials, Newtown, CT, USA) operating at 20 kHz frequency and 200 W power, with direct probe immersion into the sample solution for 30 min at 45 °C. The probe-assisted ultrasonication generated more intense cavitation and localized shear forces compared with UBE, promoting efficient cell wall disruption and enhanced mass transfer of piperine into the solvent. After extraction, the solutions were filtered through Whatman No. 1 filter paper to remove solid residues. The filtrates were analyzed by UV–visible spectrophotometry (Shimadzu, Kyoto, Japan) at 342 nm, the characteristic absorption maximum of piperine. To determine the piperine concentration (Cₚ) in each extract, a calibration curve was constructed using standard solutions of pure piperine (1–100 µg/mL) dissolved in methanol. The calibration curve followed the linear regression equation:

(1) $$C_{p}=\frac{A_{sample}-b}{m}\;$$

where *A*ₛₐₘₚₗₑ is the absorbance of the test sample, *m* is the slope, and *b* is the intercept of the calibration curve. The calculated Cₚ values (in µg/mL) were subsequently converted to mg/mL and used to determine the extraction yield as follows:

(2) $$\mathrm{Yield}\;(\mathrm{mg}/g)=\frac{C_{p}\times V_{s}}{M_{s}}\;$$

where Cₚ is the piperine concentration in the extract (mg/mL), Vₛ is the volume of solvent used (mL), and Mₛ is the mass of PNL seed powder (g). These yield values were subsequently used for the RSM optimization study.

### 2.5. RSM Optimization of Piperine Extraction Using UBE and UPE

#### 2.5.1. One-Factor Preliminary Experiments (OFAT)

Preliminary OFAT experiments were performed to determine suitable parameter ranges for subsequent RSM optimization of piperine extraction using both UBE and UPE. The factors examined included liquid-to-solid ratio, extraction time, temperature, and water content in NADES. For each test, 1.0 g of PNL seed powder was extracted with 15 mL of the selected NADES, and the filtrates were analyzed by UV–vis spectrophotometry at 342 nm. Piperine yield was calculated using Equation (2).

During each single-factor study, the remaining parameters were held constant to isolate the effect of the tested variable.

UBE: The liquid-to-solid ratio (10–30 mL/g) was examined while keeping extraction time (45 min), temperature (45 °C), and water content (20%) constant. Extraction time (15–75 min) was tested at a fixed liquid-to-solid ratio (25 mL/g), temperature (45 °C), and water content (20%). Extraction temperature (35–55 °C) was varied with liquid-to-solid ratio (25 mL/g), time (45 min), and water content (20%) constant. Finally, water content (10–50%) was investigated while keeping liquid-to-solid ratio (25 mL/g), time (45 min), and temperature (45 °C) unchanged (Table 2).

UPE: The liquid-to-solid ratio (15–35 mL/g) was tested while keeping extraction time (50 min), temperature (50 °C), and water content (30%) constant. Extraction time (20–80 min) was varied at a fixed liquid-to-solid ratio (30 mL/g), temperature (50 °C), and water content (30%). Extraction temperature (40–60 °C) was examined while maintaining liquid-to-solid ratio (30 mL/g), time (50 min), and water content (30%). Lastly, water content (20–60%) was studied with liquid-to-solid ratio (30 mL/g), time (50 min), and temperature (50 °C) constant (Table 2). The differences in parameter ranges between UBE and UPE were based on their distinct energy transfer mechanisms: the probe system provides direct, intense cavitation, requiring slightly higher temperature and time ranges for consistent comparison and solvent stability. Conversely, UBE involves indirect, uniform energy distribution, demanding lower operational ranges to avoid degradation or solvent overheating.

#### 2.5.2. RSM Experiments of UBE- and UPE-Piperine

The most promising ranges identified from the OFAT studies were used for Box–Behnken Design (BBD) experiments to optimize extraction yield. Each factor was evaluated at three levels (−1, 0, +1), and all possible interactions and quadratic effects were assessed. The same extraction and analysis procedures described above were followed. The experimental data were subjected to regression modeling to predict optimal conditions for maximum piperine yield (Table 3 and Table 4).

### 2.6. Isolation and Purification of Piperine

After extraction of piperine using the optimized UBE and UPE conditions, the crude extract was concentrated under reduced pressure at 45 °C for approximately 25–30 min using a rotary evaporator until the solvent volume was reduced to a thick, viscous residue, indicating the removal of most of the NADES solvent. The concentrated extract was then subjected to liquid–liquid extraction with dichloromethane to separate piperine from polar impurities. The dichloromethane layer containing piperine was collected and washed several times with distilled water to remove any residual NADES components. The organic layer was then dried over anhydrous sodium sulfate and filtered. The solvent was evaporated under reduced pressure to obtain crude piperine. Purification of the crude piperine was carried out by recrystallization using ethanol as a solvent. The resulting piperine crystals were collected by filtration, washed with cold ethanol to remove remaining impurities, and dried under vacuum at room temperature until a constant weight was achieved. The purified piperine was then used for further characterization.

### 2.7. Characterization of Purified Piperine

The purified piperine samples obtained from both optimized UBE and UPE processes were characterized to confirm their chemical identity, purity, structure, and morphology, enabling a comparative evaluation of the two extraction methods.

#### 2.7.1. Confirm Chemical Identity and Purity

TLC was performed to confirm the presence and assess the chemical identity and purity of Piperine by comparing the Rf values of UBE and UPE samples with the standard. Silica gel 60 F254 aluminum plates (Merck, Darmstadt, Germany) served as the stationary phase, and chloroform:methanol (9:1 *v*/*v*) was the mobile phase. A small amount of each sample and standard piperine were dissolved in ethanol and spotted on the plates. Visualization of the TLC spots was performed under normal light.

#### 2.7.2. Melting Point Determination

Melting point analysis was conducted to assess physical purity and confirm the identity of piperine by comparing observed melting points with literature values. The purified samples from UBE and UPE were finely powdered and packed into capillary tubes. Melting points were measured using a Stuart SMP10 digital melting point apparatus (Stuart, London, UK), and compared with the literature value, as impurities typically broaden or lower the melting range.

#### 2.7.3. Verify Characteristic Absorbance and Purity

The identity and purity of piperine were confirmed by recording the characteristic UV absorbance spectrum from 200 to 600 nm using a Shimadzu UV-1800 spectrophotometer (Shimadzu, Japan). Samples from UBE and UPE were dissolved in ethanol to a suitable concentration, and their spectra were compared for the characteristic absorbance peak at 342 nm and absence of extra peaks.

#### 2.7.4. Identify Functional Groups

FTIR was applied to identify functional groups characteristic of piperine. Each UBE and UPE sample were prepared by directly applying the powdered material onto the PerkinElmer Spectrum Two FTIR spectrometer (PerkinElmer, Waltham, MA, USA) in ATR mode, and spectra were recorded over the range of 4000–400 cm^−1^ to confirm the molecular structure.

#### 2.7.5. Quantify Piperine Concentration

HPLC was performed to precisely quantify the piperine content in UBE- and UPE-derived samples, facilitating a direct comparison of extraction efficiency between both methods. The analysis was carried out using an Agilent 1260 Infinity HPLC system (Agilent Technologies, USA) equipped with a HiQ Sil C18W column (250 × 4.6 mm, 5 μm). The mobile phase comprised methanol and water in a 70:30 (*v*/*v*) ratio, delivered at a flow rate of 1.0 mL/min under isocratic conditions. The detection wavelength was set at 342 nm. Each sample (1 mg/mL) was prepared by dissolving the purified extract in methanol, followed by filtration through a 0.22 μm PTFE syringe filter before injection. The injection volume was 20 μL. Quantification was achieved using a calibration curve generated from standard piperine solutions of known concentrations (10–100 μg/mL). Data acquisition and processing were performed using Agilent OpenLAB CDS software (version 2.4).

#### 2.7.6. Compare Chemical Profiles and Semi-Quantitative Content

HPTLC was used to provide a rapid, semi-quantitative assessment of piperine content and purity in UBE and UPE-derived samples. Accurately weighed samples (5 mg/mL) were dissolved in ethanol and filtered through a 0.22 μm syringe filter. Aliquots (5 μL) were applied as 8 mm bands on silica gel 60 F254 aluminum plates (Merck, Germany) using a CAMAG Linomat 5 applicator. The plates were developed in a twin-trough chamber using chloroform: methanol (9:1, *v*/*v*) as the mobile phase. After development, plates were dried and scanned densitometrically at 342 nm using a CAMAG TLC Scanner 4 with WinCATS software (version 1.4.8).

#### 2.7.7. Determine Crystalline Structure

XRD analysis was conducted to determine the crystalline structure and phase purity of piperine obtained from UBE and UPE. Powdered samples were analyzed using a Rigaku MiniFlex 600 X-ray diffractometer (Rigaku, Tokyo, Japan) with Cu-Kα radiation (λ = 1.5406 Å), operating at 40 kV and 15 mA. Diffraction patterns were recorded in the range of 5–80° (2θ) with a step size of 0.02° and a scan rate of 2°/min. Data were processed using Rigaku PDXL software (Version 2.0) to identify characteristic diffraction peaks corresponding to crystalline piperine.

#### 2.7.8. Confirm Molecular Vibrations and Structure

Raman spectroscopy was used to confirm molecular vibrations and structural features of piperine. Spectra were acquired using a Renishaw InVia Raman Microscope (Renishaw, UK) equipped with a 785 nm laser source, over the spectral range of 400–1800 cm^−1^ with a spectral resolution of 1 cm^−1^. Each powdered sample (UBE and UPE) was placed on a glass slide and analyzed directly without further treatment to prevent degradation or contamination.

#### 2.7.9. Observation of Surface Morphology and Particle Size

Surface morphology and crystal size of piperine were examined using a JEOL JSM-IT500 scanning electron microscope (JEOL, Japan). Powdered samples were mounted on aluminum stubs using carbon tape, sputter-coated with a thin layer of gold using a Quorum SC7620 sputter coater, and analyzed at an accelerating voltage of 15 kV. Images were captured at different magnifications to assess morphological differences between UBE- and UPE-derived piperine.

#### 2.7.10. Elemental Composition Analysis

Energy-dispersive X-ray spectroscopy (EDX) was performed in conjunction with SEM using an Oxford Instruments X-Max detector (Oxford Instruments, UK) to determine the elemental composition and purity of piperine samples. Spectra were acquired at 15 kV accelerating voltage with a 60 s live time. Data were analyzed using Oxford INCA software (Version 5.05) to determine the weight (%) and atomic (%) of detected elements.

#### 2.7.11. Analysis of Proton Chemical Structure

^1^H NMR spectroscopy was performed to confirm the proton chemical structure of purified piperine. Samples were dissolved in deuterated dimethyl sulfoxide (DMSO-d_6_, 10 mg/mL) and analyzed using a Bruker Avance III 400 MHz spectrometer (Bruker, Germany). Spectra were recorded at 25 °C, with chemical shifts expressed in ppm relative to tetramethylsilane (TMS) as the internal standard.

#### 2.7.12. Analysis of Carbon Chemical Structure

^13^C NMR spectroscopy was used to confirm the carbon skeleton of piperine using the same Bruker Avance III 400 MHz spectrometer and DMSO-d_6_ solvent. Spectra were acquired at 100 MHz under identical experimental conditions as ^1^H NMR, and the chemical shifts were reported in ppm relative to TMS.

### 2.8. In Vitro Studies

The in vitro studies were conducted to comparatively evaluate the biological activities of purified piperine obtained from the optimized UBE and UPE processes using NADES as green solvents.

#### 2.8.1. Cytotoxicity of UBE- and UPE-Derived Piperine in C2C12 Cells

The cytotoxicity of purified piperine samples obtained from UBE and UPE was evaluated in C2C12 myoblast cells using the MTT assay [43]. C2C12 cells were cultured in DMEM, supplemented with 10% fetal bovine serum (FBS) and 1% penicillin–streptomycin under standard culture conditions (37 °C, 5% CO_2_, humidified atmosphere). Prior to cell treatment, the piperine samples were sterilized by filtration through 0.22 µm syringe filters and diluted in complete culture medium to obtain the desired concentrations. A total of 8000 cells per well were seeded in 96-well plates and allowed to adhere overnight. Cells were treated in triplicate with various concentrations of piperine (0.19–100 µg/mL) for 48 h, while DMSO served as the vehicle control. Each experiment was performed in three independent biological replicates to ensure statistical reliability. Following treatment, MTT solution (5 mg/mL) was added to each well, and the plates were incubated to allow for the formation of formazan crystals. These crystals were subsequently dissolved in DMSO, and the absorbance was measured at 570 nm using a BioTek microplate reader (BioTek Instruments, USA). Cell viability (%) was calculated using the following formula:

(3) $$Cell\;viability\left(\%\right)=\frac{Optical\;density\;of\;piperine\;treated\;cells}{Optical\;density\;of\;control\;cells}\times 100$$

IC_50_ values were determined from the dose–response curves, enabling a comparative evaluation of the cytotoxic effects of piperine extracted via UBE and UPE.

#### 2.8.2. Anticancer Activity Assessment

The anticancer potential of purified piperine obtained from UBE and UPE was evaluated using MCF-7 human breast cancer and HT-29 colon cancer cell lines via the MTT assay [12]. Cells were maintained in DMEM containing 10% FBS and 1% penicillin–streptomycin and cultured at 37 °C in a humidified 5% CO_2_ incubator. Piperine samples were sterilized using 0.22 µm syringe filters and freshly diluted in the culture medium before each treatment. Cells were seeded in 96-well plates at a density of 2 × 10^4^ cells per well and incubated for 24 h to allow adherence. Subsequently, cells were treated with varying concentrations of piperine (15, 30, and 45 µg/mL) for 48 h. Doxorubicin served as a positive control, and DMSO-treated cells were used as a negative control. After treatment, MTT solution was added, formazan crystals were dissolved in DMSO, and absorbance was measured at 570 nm, as described above. All treatments were performed in triplicate wells for each concentration, and the entire experiment was repeated three times independently IC_50_ values were calculated for each sample to quantitatively compare the anticancer activity of UBE- and UPE-derived piperine against MCF-7 and HT-29 cell lines.

#### 2.8.3. Antioxidant Activity

##### In Vitro Antioxidant Assays

The antioxidant potential of purified piperine obtained from UBE and UPE was evaluated using DPPH, ABTS, FRAP, and TAC assays. Test samples were dissolved in ethanol, filtered through 0.22 µm syringe filters for sterilization, and prepared at concentrations of 2–10 mg/mL. All measurements were performed in triplicate to ensure reproducibility. Radical scavenging activity and overall antioxidant capacity were calculated relative to the respective reference standards. Detailed assay conditions, including reagent preparation, incubation times, and detection wavelengths, are summarized in Table 5. Percentage scavenging or antioxidant activity was determined using the following formula:

(4) $$\%Scavenging=\frac{A_{control}{\;-\;A}_{test}}{A_{control}}\times 100\;$$

##### Intracellular ROS and RNS Evaluation in THP-1 and RAW 264.7 Cells

To assess the intracellular antioxidant effects of piperine from UBE and UPE, THP-1 and RAW 264.7 cells were cultured in RPMI-1640 and DMEM media, respectively, supplemented with 10% FBS and 1% penicillin–streptomycin at 37 °C in a humidified 5% CO_2_ atmosphere. Piperine samples were freshly diluted in sterile culture medium and filtered through 0.22 µm syringe filters prior to treatment. Cells were seeded in black, clear-bottom 96-well plates and incubated with piperine at concentrations ranging from 5 to 45 µg/mL for 30 min at 37 °C. Intracellular ROS levels were measured by loading cells with 15 µM DCFH-DA in serum-free medium, followed by a 35 min incubation in the dark to allow oxidation-dependent fluorescence formation. Fluorescence, proportional to ROS levels, was recorded using a microplate reader (excitation: 488 nm; emission: 525 nm). Intracellular RNS levels were assessed using 15 µM BBoxiProbe™ R21F, which generates fluorescence upon reaction with reactive nitrogen species. Fluorescence intensity was recorded under the same parameters as ROS detection. Each experiment included triplicate wells and three independent biological replicates, and results were expressed relative to untreated control cells [48].

### 2.9. Statistical Analysis

All experimental measurements are reported as the mean ± standard deviation (SD). Differences among groups were evaluated using either one-way or two-way analysis of variance (ANOVA), depending on the experimental design. Statistical computations and figure generation were performed using GraphPad Prism 10.4.2 (GraphPad Software, La Jolla, CA, USA) and Origin 2018 (64-bit).

## 3. Results and Discussion

### 3.1. Efficacy of Piperine Extraction Using NADES

The efficiency of piperine extraction was systematically evaluated using six different NADESs. The NADES compositions consisted of various HBA and HBD in different molar ratios, selected based on their reported ability to enhance solubility of bioactive compounds and facilitate green extraction processes. Previous studies have demonstrated that NADES exhibit excellent solvent properties due to their tunable polarity, viscosity, and hydrogen bonding capacity, making them ideal for bioactive compound extraction [49,50]. After preparation, the NADES mixtures were cooled to room temperature and stored in airtight containers. Their stability was confirmed by the absence of phase separation or precipitation after standing for at least 24 h under ambient conditions, consistent with earlier reports [51]. Water was deliberately added to NADES to reduce viscosity and improve mass transfer during extraction, thereby enhancing the solubilization of piperine, as supported by previous research [52]. The role of water content is critical in modifying the physicochemical properties of NADES, facilitating better penetration into plant matrices and disrupting cell walls [53]. The piperine yield was determined by UV-Vis spectrophotometry at 342 nm, which is the characteristic absorbance peak for piperine, one of the most abundant bioactive compounds in PNL [54]. A calibration curve was generated using six different concentrations of Piperine to ensure accurate quantification.

#### 3.1.1. Piperine Yield from UBE

The piperine yield obtained using UBE varied significantly depending on the NADES composition, ranging from 9.83 mg/g to 25.67 mg/g (Figure S1A). Among the NADES tested, NADES-5 (Choline Chloride: Glycerin + Urea, 1:1:1) achieved the highest piperine yield of 25.67 mg/g. This superior performance is likely due to the ternary NADES system providing an optimal combination of polarity, reduced viscosity, and enhanced hydrogen bonding interactions, which collectively improve piperine solubilization and facilitate efficient mass transfer from the plant matrix. In contrast, NADES-4 (Choline Chloride: Malic Acid, 1:1) exhibited the lowest piperine yield (9.83 mg/g), probably due to its relatively higher viscosity and lower polarity, which hinder effective interaction between the solvent and piperine molecules during extraction. These results clearly demonstrate that NADES composition plays a pivotal role in the extraction efficiency of UBE, aligning with previous findings that emphasize the critical impact of solvent physicochemical properties such as hydrogen bonding capacity and viscosity on bioactive compound recovery [52,53].

#### 3.1.2. Piperine Yield from UPE

Piperine extraction using UPE consistently resulted in higher yields compared to UBE, with values ranging from 24.81 mg/g to 49.97 mg/g (Figure S1B). Again, NADES-5 demonstrated the highest extraction efficiency, delivering a maximum piperine yield of 49.97 mg/g. The improved performance of UPE can be explained by the intense cavitation and strong localized shear forces generated by the probe system, which more effectively disrupt plant cell walls and enhance the mass transfer of piperine into the NADES solvent. Across all tested NADES, UPE increased piperine yield by approximately 50–60% compared to UBE, strongly supporting the primary hypothesis of this study. Similarly to UBE, NADES-4 resulted in the lowest yield (24.81 mg/g), confirming its limited ability to solubilize piperine under probe-assisted ultrasonication. Based on these systematic results, NADES-5 was selected for further process optimization using RSM due to its superior preliminary extraction performance in both UBE and UPE.

### 3.2. One-Factor Preliminary Experiments of UBE and UPE

The influence of individual extraction parameters on piperine yield using both UBE and UPE was systematically evaluated by varying one factor at a time while keeping the others constant, to identify suitable ranges for subsequent RSM optimization.

#### 3.2.1. Effect of Liquid-to-Solid Ratio

**Figure 1:**
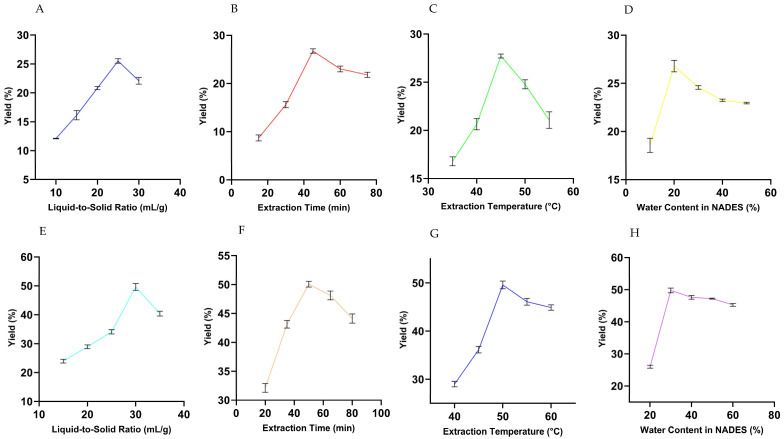
Effect of individual extraction parameters on piperine yield using UBE and UPE. Data represent the mean ± SD of three independent experiments performed in triplicate. (**A**,**E**) Liquid-to-solid ratio, (**B**,**F**) Extraction time, (**C**,**G**) Extraction temperature, and (**D**,**H**) Water content in NADES.

As shown in Figure 1A,E the piperine yield exhibited a clear dependence on the liquid-to-solid ratio for both UBE and UPE. In UBE, the yield increased from 12.10 mg/g to a maximum of 25.51 mg/g as the ratio rose from 10 to 25 mL/g, but further increasing to 30 mL/g led to a slight decline to 22.10 mg/g. This trend suggests that a ratio of 25 mL/g provided an optimal balance between sufficient solvent availability for solubilization and efficient mass transfer while avoiding excessive dilution that could hinder extraction efficiency [30]. For UPE, increasing the liquid-to-solid ratio from 15 mL/g to 30 mL/g significantly enhanced piperine yield, reaching a maximum of 49.60 mg/g, followed by a slight decrease to 40.38 mg/g at 35 mL/g. This indicates that 30 mL/g was optimal, offering efficient solubilization and diffusion without unnecessary dilution of the solvent system [36].

#### 3.2.2. Effect of Extraction Time

As illustrated in Figure 1B,F, extraction time strongly influenced the yield of piperine in both UBE and UPE. In UBE, the yield increased steadily up to 45 min, reaching a peak of 26.74 mg/g, beyond which a decline was observed at 60 and 75 min. This reduction may be due to the degradation of piperine or decreased extraction efficiency caused by solvent saturation or other limiting factors [30]. In UPE, a similar pattern was observed the yield increased with time and reached its maximum (50.06 mg/g) at 50 min, after which it slightly declined, suggesting that prolonged sonication offered no further benefit and might even cause mild degradation of piperine or reduced solvent efficacy [55]. Hence, 45 min for UBE and 50 min for UPE were considered the optimal extraction durations.

#### 3.2.3. Effect of Extraction Temperature

Figure 1C,G demonstrate the effect of extraction temperature on piperine yield. For UBE, the yield increased with temperature and peaked at 45 °C with an average of 27.72 mg/g. Below this temperature, the extraction rate was limited by higher solvent viscosity and reduced molecular mobility, whereas above 45 °C, the yield declined, likely due to degradation of thermolabile components or decreased solvent efficiency [14]. Similarly, in UPE, the yield rose with increasing temperature, achieving a maximum of 49.58 mg/g at 50 °C. Further increases to 55 °C and 60 °C slightly reduced the yield, which could be attributed to piperine degradation or reduced solvent efficacy at elevated temperatures [56]. Therefore, 45 °C and 50 °C were identified as the optimal extraction temperatures for UBE and UPE, respectively.

#### 3.2.4. Effect of Water Content in NADES

As shown in Figure 1D,H, the water content in NADES significantly influenced the extraction yield. In UBE, varying the water content from 10% to 50% revealed that the highest piperine yield (26.79 mg/g) was obtained at 20% water content. This moderate water proportion effectively lowered the viscosity of NADES, facilitating better solvent penetration into the plant matrix and enhancing mass transfer. Increasing the water content beyond 20% did not improve yield and caused a slight decline due to excessive dilution of the solvent [57]. In UPE, the optimal water content was found to be 30%, producing the highest yield of 49.74 mg/g. Here, moderate water addition reduced viscosity and improved solvent accessibility, whereas further increases to 40–60% resulted in minor decreases in yield, suggesting reduced solvent performance due to over-dilution [58]. Thus, 20% and 30% water contents were identified as the optimal levels for UBE and UPE, respectively.

The optimized OFAT conditions established for UBE (25 mL/g, 45 min, 45 °C, 20% water) and UPE (30 mL/g, 50 min, 50 °C, 30% water) served as the basis for subsequent multivariate optimization using Response Surface Methodology (RSM). Optimization of the fitted quadratic models yielded the following RSM-predicted optima. For UBE, the stationary point was at A = 26.82 mL/g, B = 47.44 min, C = 44.81 °C, D = 22.41%, giving a predicted piperine yield of 27.14 mg/g. For UPE, the stationary point was at A = 28.12 mL/g, B = 47.26 min, C = 44.58 °C, D = 29.85%, giving a predicted yield of 53.65 mg/g. The UPE temperature optimum (44.58 °C) is approximately 0.4 °C below the lower experimental level (45 °C) and may therefore be practically implemented as 45 °C. Compared with the OFAT-derived conditions, RSM provided small but meaningful refinements: for UBE, the predicted yield increased slightly (~+0.4 mg/g), while for UPE, RSM predicted a larger improvement (~+2.6 mg/g), indicating a greater benefit from exploring factor interactions and curvature for the probe-assisted method.

### 3.3. BBD and RSM Optimization of UBE-Piperine

In the case of UBE, the optimization study was carried out using a four-factor, three-level BBD. The selected independent variables were liquid-to-solid ratio (A), extraction time (B), extraction temperature (C), and water content in NADES (D), while the response variable (R1) was defined as the yield of piperine. A total of 29 experimental runs were performed according to the design matrix, and the corresponding extraction yields are summarized in Table 6. This design enabled the systematic evaluation of both the individual and interactive effects of the four process variables on piperine recovery and provided the experimental basis for developing a predictive regression model.

#### 3.3.1. Fitting of the UBE-Piperine Regression Equation and Analysis of Variance

To comprehensively evaluate the combined effects of the four factors on piperine yield, the experimental data obtained from the BBD (Table 6) were fitted to a second-order polynomial model. The resulting quadratic regression equation was expressed as follows:

(5)                    R_1_ = −214.836667 + 4.243333A + 1.315667B + 6.229333C + 1.277333D                   −0.063067A^2^ − 0.011841B^2^ − 0.064567C^2^ − 0.013267D^2^ −0.006333AB − 0.008000AC − 0.009000AD −0.000333BC − 0.000333BD − 0.009500CD

where R_1_ is the piperine yield (mg/g); A = solid–liquid ratio (mL/g), B = extraction time (min), C = extraction temperature (°C), and D = water content (%).

The ANOVA results (Supplementary Table S1) demonstrated that the regression model was highly significant (F = 88.02, *p* < 0.0001). The coefficients of determination (R^2^ = 0.9796) and adjusted R^2^ (0.9592) confirmed the strong explanatory power of the model. The predicted R^2^ (0.8851) was in reasonable agreement with the adjusted R^2^, suggesting good model reliability. The lack-of-fit test was found to be non-significant (*p* > 0.05), indicating that the quadratic model adequately fitted the experimental data without systematic deviation. From the factor significance analysis, all four linear terms (A, B, C, D) and the quadratic terms (A^2^, B^2^, C^2^, D^2^) were highly significant (*p* < 0.0001), highlighting strong curvature effects in the response surface. Significant interaction effects were observed for A × B, A × D, and C × D (*p* < 0.05), while the interactions A × C, B × C, and B × D were not significant (*p* > 0.05). Diagnostic parameters (Supplementary Table S2) further validated the adequacy of the model: low standard deviation (0.398), small coefficient of variation (1.68%), high Adeq Precision (17.82), and acceptable PRESS values confirmed good predictive accuracy. Collectively, these results demonstrate that the model can reliably predict piperine yield under UBE conditions and is suitable for process optimization [23,39].

#### 3.3.2. Interactive Effects of UBE Process Variables on Piperine Yield

**Figure 2:**
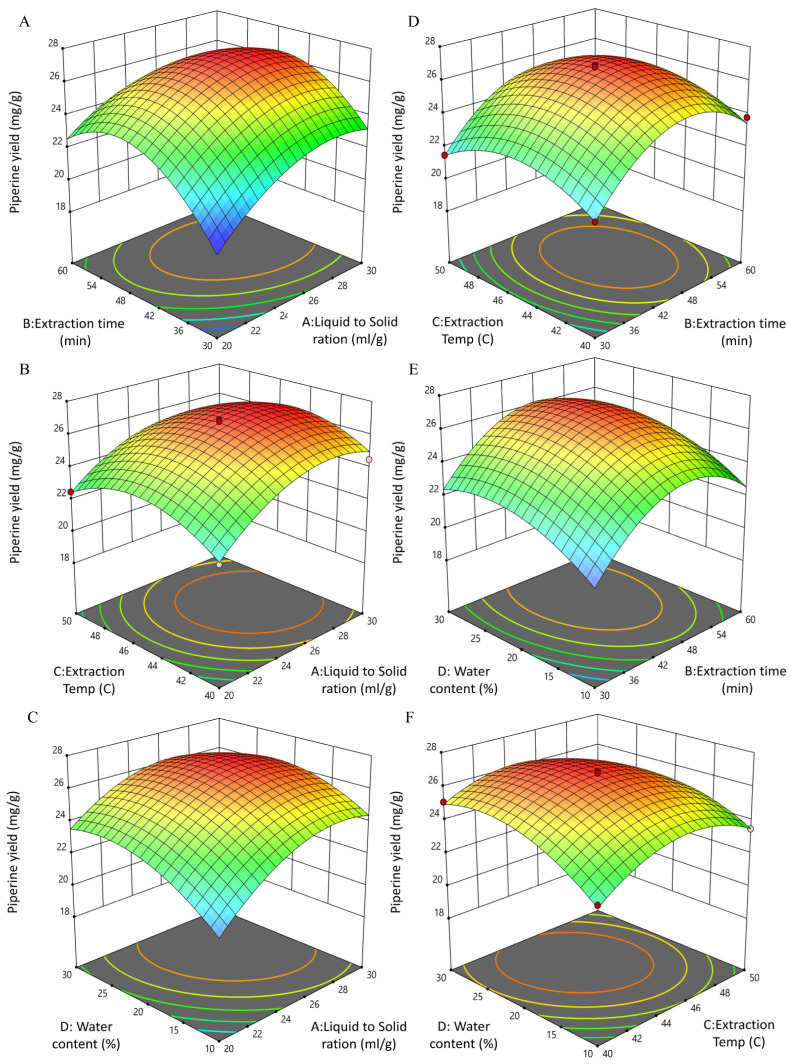
Three-dimensional response surface plots showing the interactive effects of extraction parameters on piperine yield during UBE. Interactions include: (**A**) liquid-to-solid ratio × extraction time, (**B**) liquid-to-solid ratio × temperature, (**C**) liquid-to-solid ratio × water content, (**D**) extraction time × temperature, (**E**) extraction time × water content, and (**F**) temperature × water content. The plots were generated using Design-Expert software (version 13).

The three-dimensional response surface and contour plots (Figure 2; Supplementary Figure S2) were generated to visualize the pairwise interactions of A: Liquid-to-solid ratio, B: Extraction time, C: Temperature, and D: Water content on piperine yield. The ANOVA results (Supplementary Table S1) confirmed that three interactions A × B, A × D, and C × D were statistically significant (*p* < 0.05), while the others (A × C, B × C, B × D) were not significant (*p* > 0.05). The interaction between A and B (Figure 2A; Supplementary Figure S2A) showed a convex surface and elliptical contours, indicating that piperine yield increased as both liquid-to-solid ratio and extraction time rose to intermediate levels. However, excessive increases in either factor did not further enhance the yield, consistent with the quadratic regression model.

Similarly, the A × D interaction (Figure 2C; Supplementary Figure S2C) revealed that moderate water content in NADES, when combined with a balanced solvent loading, improved extraction performance, while too little or too much water decreased the yield. The C × D interaction (Figure 2F; Supplementary Figure S2F) also showed significance, suggesting that water content modulated the effect of temperature: optimal yields were observed at mid-range temperature and water levels, whereas extreme combinations reduced efficiency. In contrast, the A × C (Figure 2B; Supplementary Figure S2B), B × C (Figure 2D; Supplementary Figure S2D), and B × D interactions were not significant. Their response surfaces appeared relatively flat with nearly circular contour plots, indicating that the effect of one factor was largely independent of the other.

These results are consistent with the lack of statistical significance in the ANOVA (*p* > 0.05). Overall, the significant interactions highlight the importance of balancing solvent loading, extraction time, temperature, and water content in combination, rather than optimizing them individually. The downward-opening response surfaces across all models confirm the quadratic nature of the regression and the presence of well-defined optimum conditions for maximizing piperine yield under UBE [36,39].

### 3.4. BBD and RSM Optimization of UPE-Piperine

For UPE, the optimization study was conducted using a four-factor, three-level BBD. The independent variables chosen included liquid-to-solid ratio (A), extraction time (B), extraction temperature (C), and water content in NADES (D), with piperine yield (R1) serving as the response variable. A total of 29 experimental runs were carried out according to the design matrix, and the resulting yields are presented in Table 7. This approach allowed for a systematic assessment of both the individual and interactive effects of the four factors on piperine extraction and provided a solid foundation for constructing a predictive regression model.

#### 3.4.1. Fitting of the UPE-Piperine Regression Equation and Analysis of Variance

The Box–Behnken experimental data for UPE (Table 7) were fitted to a second-order polynomial model to assess the combined effects of extraction parameters on piperine yield. The quadratic regression equation was obtained as follows:

(6)                    R_2_ = −232.541667 + 5.113333A + 1.414667B + 7.014667C + 1.642000D                    −0.072133A^2^ − 0.012267B^2^ − 0.072067C^2^ − 0.014533D^2^ −0.007667AB − 0.008667AC − 0.010333AD −0.000667BC − 0.000333BD − 0.010500CD

where R_2_ is the piperine yield (mg/g); A = solid–liquid ratio (mL/g), B = extraction time (min), C = extraction temperature (°C), and D = water content (%).

The ANOVA (Supplementary Table S3) confirmed that the quadratic model was highly significant (F = 95.21, *p* < 0.0001). The coefficients of determination (R^2^ = 0.9817) and adjusted R^2^ (0.9629) indicated that the model effectively explained variability in piperine yield. The predicted R^2^ (0.9012) was consistent with the adjusted R^2^, suggesting strong predictive reliability. The lack-of-fit was non-significant (*p* > 0.05), confirming that the model provided an adequate representation of the experimental data. From the factor analysis, all four main effects (A, B, C, D) and their quadratic terms were highly significant (*p* < 0.0001), confirming nonlinear influences of each parameter. Significant interactions were observed for A × B, A × D, and C × D (*p* < 0.05), while other interactions (A × C, B × C, B × D) were not statistically significant. Model validation (Supplementary Table S4) demonstrated strong predictive performance: a small standard deviation (0.412), low coefficient of variation (0.89%), high Adeq Precision (19.52), and acceptable PRESS value supported the adequacy of the model [23,36,39].

#### 3.4.2. Interactive Effects of UPE Process Variables on Piperine Yield

**Figure 3:**
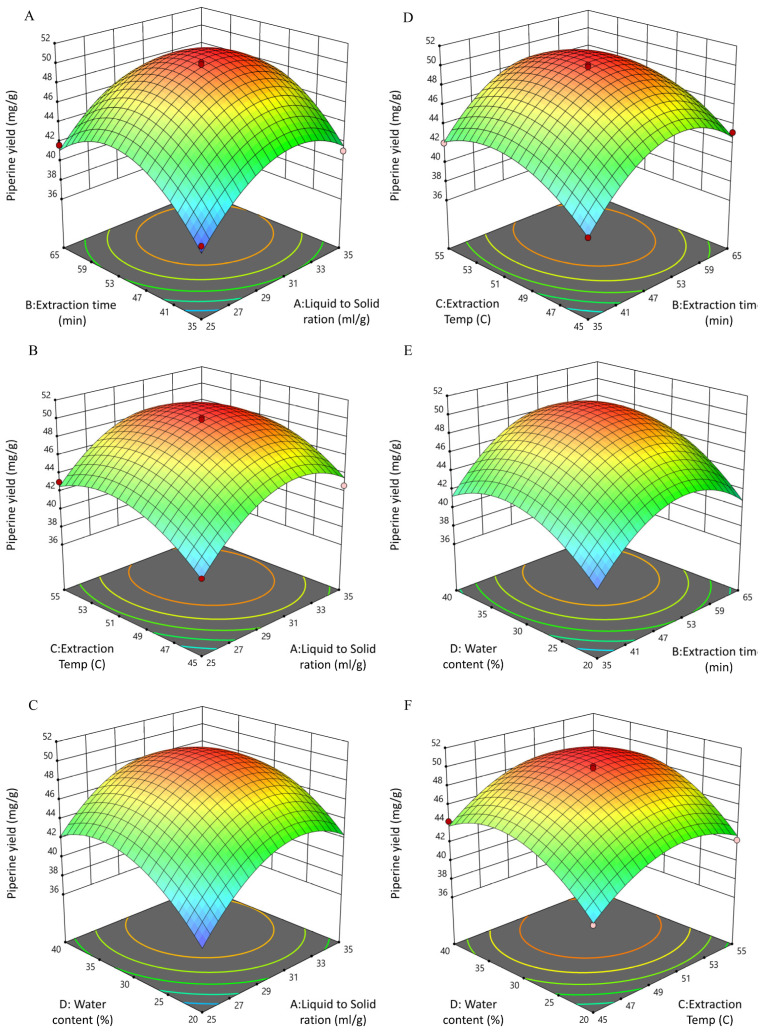
Three-dimensional response surface plots showing the interactive effects of extraction parameters on piperine yield during UPE. Interactions include: (**A**) liquid-to-solid ratio × extraction time, (**B**) liquid-to-solid ratio × temperature, (**C**) liquid-to-solid ratio × water content, (**D**) extraction time × temperature, (**E**) extraction time × water content, and (**F**) temperature × water content. The plots were generated using Design-Expert software (version 13).

The three-dimensional response surface plots and corresponding contour maps for UPE were generated to visualize the interactions among the four extraction parameters: liquid-to-solid ratio (A), extraction time (B), extraction temperature (C), and water content in NADES (D) (Figure 3A–F; Supplementary Figure S3A–F). These plots provide a graphical interpretation of the regression model, highlighting how paired variables influence piperine yield while keeping the other factors constant at their center levels. The interaction between A × B was significant (*p* < 0.05), as supported by the ANOVA results (Supplementary Table S3). The surface plot for A × B revealed a moderately steep curvature, while the contour lines exhibited an elliptical pattern, confirming the interactive effect of these two factors on yield. Similarly, the A × D interaction was also significant (*p* < 0.05). The corresponding response surface displayed a noticeable slope with oval-shaped contours, suggesting that optimal tuning of solvent composition and ratio contributes to maximizing extraction efficiency. Another significant interaction was observed between C × D, with the 3D plot showing distinct curvature and the contours forming elliptical shapes, again validating the statistical significance (*p* < 0.05). In contrast, the interactions A × C, B × C, and B × D were not statistically significant (*p* > 0.05). Their response surfaces appeared relatively flat, and the contour plots showed nearly circular lines rather than elliptical ones, indicating weak or negligible synergistic effects between these factor pairs. Overall, the downward-facing response surfaces across significant plots confirm the existence of optimal operating points for UPE, in line with the quadratic model predictions. Importantly, compared to UBE, the UPE surfaces appeared steeper and better defined, particularly for extraction time and temperature, consistent with the higher regression coefficients and diagnostic statistics (R^2^ = 0.9817, Adj R^2^ = 0.9629, Pred R^2^ = 0.9012). This demonstrates that UPE not only enhances extraction yield but also offers a more robust optimization landscape than UBE. When both methods were compared, UPE showed a clear statistical advantage, providing stronger predictive accuracy, higher model adequacy, and lower experimental variation. These comparative outcomes indicate that while UBE remains reliable, UPE delivers a more precise and robust optimization model for piperine yield [23,36,39,59].

### 3.5. Isolation and Purification of Piperine Obtained from UBE and UPE

**Figure 4:**
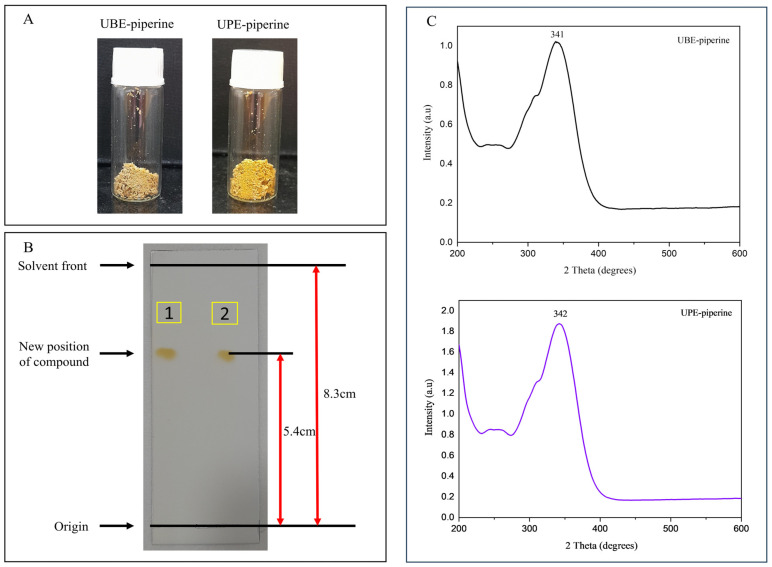
Preliminary identification and purity of isolated piperine. (**A**) Morphology of purified piperine crystals: UBE-piperine appears as light-yellow irregular aggregates, whereas UPE-piperine appears as dark-yellow aggregates. (**B**) TLC analysis of UBE- and UPE-piperine on a pre-coated silica gel TLC plate under normal light: (**1**) UBE-piperine, (**2**) UPE-piperine. (**C**) UV–vis spectra of UBE- and UPE-piperine showing characteristic absorption maxima.

The crude extracts obtained under optimized UBE and UPE conditions were successfully concentrated and subjected to liquid–liquid extraction and recrystallization, yielding pure piperine crystals. The purified compound appeared as irregular crystalline aggregates (Figure 4A). The use of dichloromethane effectively removed polar impurities, while ethanol recrystallization provided high-purity piperine suitable for further analyses [3]. Notably, the yield of purified piperine obtained from UPE was slightly higher than that from UBE, reflecting the superior extraction efficiency of the ultrasonic probe method. This difference can be attributed to the more effective cell wall disruption and solvent penetration achieved under probe-assisted ultrasonication, which facilitated greater recovery of piperine [42]. Importantly, the purification strategy employed was reproducible for both extraction methods, ensuring that subsequent characterizations were not influenced by residual NADES components or other impurities. These results confirm that the applied purification procedure was effective in isolating piperine from both UBE- and UPE-derived extracts, providing material of sufficient quality for structural, purity, and morphological characterization.

### 3.6. Characterization of Purified UBE- and UPE-Derived Piperine

The purified piperine samples obtained from optimized UBE and UPE processes were systematically characterized to confirm their chemical identity, structural integrity, purity, and morphological features. This allowed a comparative evaluation of the two extraction methods.

#### 3.6.1. TLC Analysis

TLC was employed as a preliminary technique to verify the presence of piperine and assess its purity. Both UBE- and UPE-derived piperine samples displayed a single, well-defined circular spot with an identical Rf value of 0.650 under normal light (Figure 4B). The consistency of Rf values across the samples indicates the successful isolation of piperine with minimal impurities. Moreover, the observed Rf value agrees with previously reported values, confirming the authenticity of the isolated compound [3].

#### 3.6.2. Melting Point Determination

Melting point analysis was performed to assess the physical purity of piperine and further validate its identity. The purified UBE-derived piperine exhibited a melting range of 131–132.5 °C, while UPE-derived piperine melted at 130–131.7 °C. These results closely correspond with reported literature values for pure piperine (129–130 °C) [2]. The narrow melting ranges indicate high purity, as impurities typically broaden or depress the melting point.

#### 3.6.3. UV-Vis Spectral Analysis

UV–vis spectrophotometry was utilized to confirm the presence of conjugated chromophores characteristic of piperine. Both samples were dissolved in ethanol at comparable concentrations (0.545 × 10^−5^ M), and their spectra were recorded from 200 to 600 nm. UBE-piperine showed a λmax at 341 nm, whereas UPE-piperine exhibited λmax at 342 nm (Figure 4C). The close similarity of absorption maxima confirms the chemical identity of piperine and indicates the absence of significant impurities, as no additional peaks were observed [60].

#### 3.6.4. FTIR Spectral Analysis

**Figure 5:**
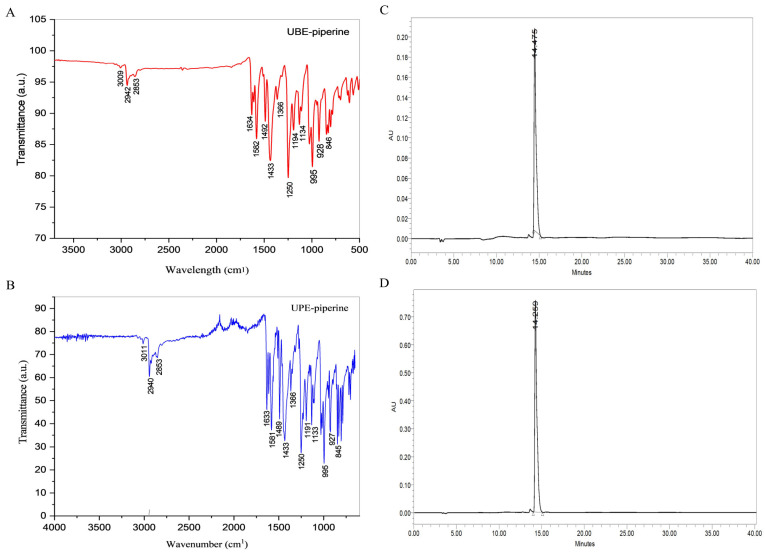
FTIR spectra of UBE-piperine crystals (**A**) and UPE-piperine crystals (**B**), and HPLC chromatograms of UBE-piperine (**C**) and UPE-piperine (**D**).

The FTIR spectra of purified piperine obtained from both UBE and UPE revealed the characteristic functional groups that confirm its chemical identity. UBE-piperine showed absorption bands at 3009 cm^−1^ (aromatic C–H stretching), 2942 and 2853 cm^−1^ (aliphatic C–H stretching), 1634 cm^−1^ (amide –CO–N– vibration), and multiple peaks at 1610, 1582, and 1492 cm^−1^ (aromatic C=C stretching). Additional peaks were observed at 1433 cm^−1^ (methylenedioxy bending), 1250 and 1194 cm^−1^ (C–O–C stretching), and 995 and 928 cm^−1^ (trans–CH=CH bending and C–O stretch) (Figure 5A). Similarly, UPE-piperine exhibited a closely matching profile with bands at 3011, 2940, 2853, 1633, 1610, 1581, 1489, 1440, 1250, 1194, 1028, 995, and 927 cm^−1^ (Figure 5B). The close overlap between both spectra indicates that the chemical structure of piperine remained unaltered regardless of the extraction technique. Minor differences in band intensities may be attributed to crystallinity variations and sample preparation rather than structural changes. These findings agree with previous vibrational assignments for pure piperine and confirm successful isolation from both methods [54].

#### 3.6.5. HPLC Chromatographic Analysis

HPLC, employed as the gold standard for purity assessment and quantification revealed that both UBE- and UPE-piperine exhibited sharp, well-resolved peaks with retention times of 14.259 min (UBE) and 14.475 min (UPE), consistent with reference piperine (Figure 5C,D) [61]. The purity, based on peak area normalization, was 98.2% for UBE-piperine and 98.7% for UPE-piperine, confirming that both approaches yielded highly purified compounds. The yield of purified piperine was 7.9% for UBE and 8.2% for UPE, with the higher recovery in UPE further supporting its greater efficiency. These yield values are consistent with those previously reported for ultrasound-assisted extraction methods [29,30]. Collectively, the findings confirm the study’s aim by demonstrating that UPE provides superior purification efficiency compared to UBE [62,63].

#### 3.6.6. HPTLC Fingerprinting Analysis

**Figure 6:**
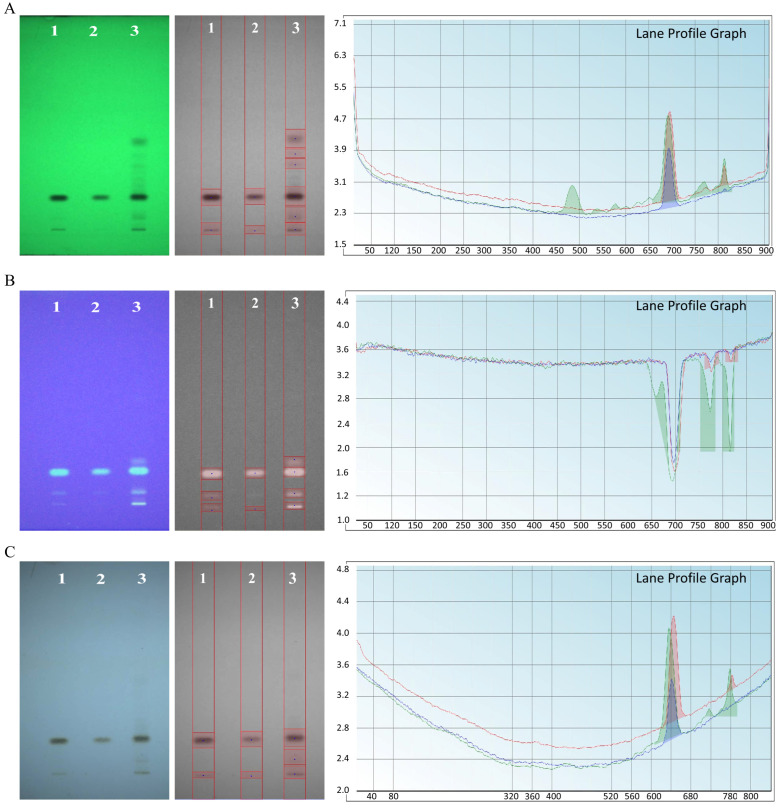
HPTLC profiles of purified piperine (UBE and UPE) and PNL extract at three detection wavelengths: (**A**) 254 nm, (**B**) 366 nm, and (**C**) 400–700 nm. In each panel, Lane 1 represents UBE-piperine, Lane 2 UPE-piperine, and Lane 3 PNL extract. Corresponding lane profile graphs display UBE-piperine 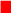, UPE-piperine (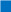), and PNL extract (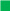).

HPTLC profiling was performed to compare the chemical fingerprints of UBE- and UPE-derived piperine with crude black pepper extract (Figure 6). At 254 nm, crude extract displayed six bands, reflecting multiple phytochemicals, whereas UBE- and UPE-piperine showed only two dominant bands each. Densitometric analysis revealed displayed volumes of 391.74 for UBE and 182.52 for UPE, with the markedly lower values for UPE indicating reduced impurity levels. At 366 nm, black pepper extract exhibited four fluorescent bands, while UBE- and UPE-piperine displayed three and two bands, respectively, with displayed volumes of 52.86 (UBE) and 0.92 (UPE). In the visible range (400–700 nm), the crude extract showed four bands, while both purified samples presented only two each, with displayed volumes of 164.35 (UBE) and 77.59 (UPE). The consistently lower displayed volumes of UPE-piperine across all detection wavelengths strongly demonstrate superior purification efficiency of UPE compared to UBE, thereby reinforcing the study aim of establishing probe-assisted extraction as a more effective approach for obtaining high-purity piperine [43].

#### 3.6.7. XRD Analysis

XRD analysis was employed to examine the crystalline structure of piperine obtained via UBE and UPE. Both samples exhibited a monoclinic crystal structure consistent with the chemical formula C_17_H_19_NO_3_, as referenced by the standard JCPDS card (No. 00-043-1627). The reference pattern reports characteristic diffraction peaks at 2θ values around 12.3°, 17.6°, 19.8°, 23.6°, 26.2°, and 28.5°, which correspond to the (110), (200), (210), (211), (220), and (310) planes, respectively.

**Figure 7:**
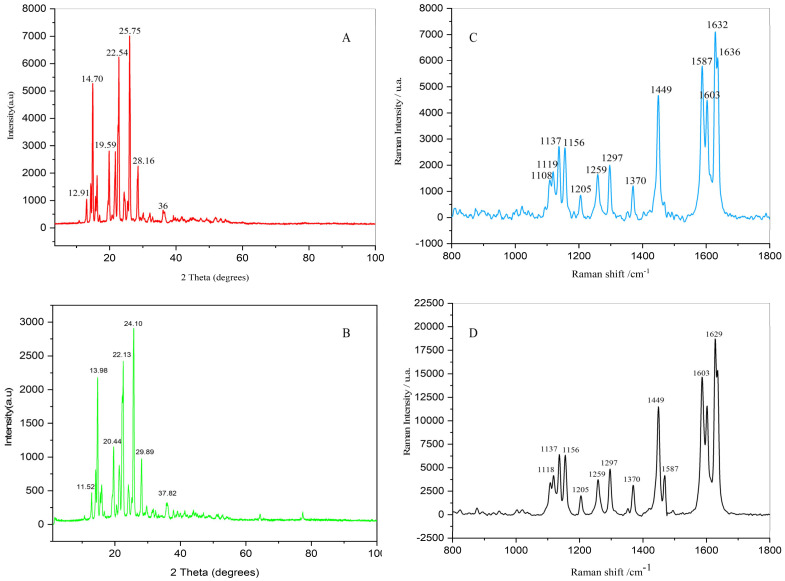
Structural and crystalline characterization of purified piperine. XRD patterns (**A**,**B**) and Raman spectra (**C**,**D**) highlighting characteristic crystalline peaks and vibrational bands, confirming the molecular structure and integrity of piperine obtained via both UBE and UPE.

The diffractograms of both UBE- and UPE-derived piperine showed reflections consistent with these standard peaks, confirming their crystalline purity. UBE-piperine exhibited prominent peaks at 2θ = 12.91° and 25.75°, with medium-intensity reflections at 14.70°, 15.94°, 19.59°, 22.54°, 28.16°, and 36.13° (Figure 7A). In contrast, UPE-piperine displayed notable peaks at 2θ = 11.52° and 24.10°, with additional reflections at 13.98°, 16.25°, 20.44°, 22.13°, 29.89°, and 37.82° (Figure 7B). The sharper and more intense reflections observed in the UPE-piperine pattern suggest a higher degree of crystallinity compared to UBE-piperine, indicating that ultrasonic probe extraction better preserves the ordered lattice structure of piperine during isolation [64]. For clearer visualization, the diffractograms have been enlarged in the 10–40° (2θ) range to emphasize the similarities and minor shifts between the UBE and UPE samples and the standard JCPDS reference [2].

#### 3.6.8. Raman Spectroscopy Analysis

Raman spectroscopy was employed to further confirm the molecular structure and vibrational characteristics of UBE- and UPE-piperine. The Raman spectrum of UBE-piperine (1108–1632 cm^−1^) showed characteristic bands: the main C=C aromatic/aliphatic stretching peak at 1632 cm^−1^, asymmetric –O=C–N stretching at 1587 cm^−1^, wagging of –CH_2_ (O–CH_2_–O) at 1449 cm^−1^, twisting of –CH_2_ in the piperidine ring at 1297 cm^−1^, and rocking/scissoring vibrations at 1259 and 1370 cm^−1^. Bands between 1108 and 1205 cm^−1^ corresponded to asymmetric C–C stretching in the piperidine ring (Figure 7C). The spectra of UPE-piperine exhibited similar prominent peaks between 1629 and 1603 cm^−1^, corresponding to aromatic and aliphatic C=C stretching vibrations. The peak at 1587 cm^−1^ was assigned to the asymmetric stretching of the –O=C–N bond. Wagging vibrations of the –CH_2_ (O–CH_2_–O) group in the phenyl methylenedioxy ring appeared at 1449 cm^−1^, while rocking and scissoring modes of –CH bonds were observed at 1370 and 1259 cm^−1^, respectively. Twisting modes of –CH_2_ bonds in the piperidine ring were detected at 1297 cm^−1^, with C–C stretching vibrations between 1156 and 1370 cm^−1^ (Figure 7D). Minor differences in intensity and peak positions between UBE and UPE are likely due to slight variations in crystallinity and sample preparation rather than structural changes. Overall, the Raman spectra of both samples closely align with literature reports, confirming the molecular integrity of piperine isolated via both extraction methods [65].

#### 3.6.9. SEM Morphological Analysis

**Figure 8:**
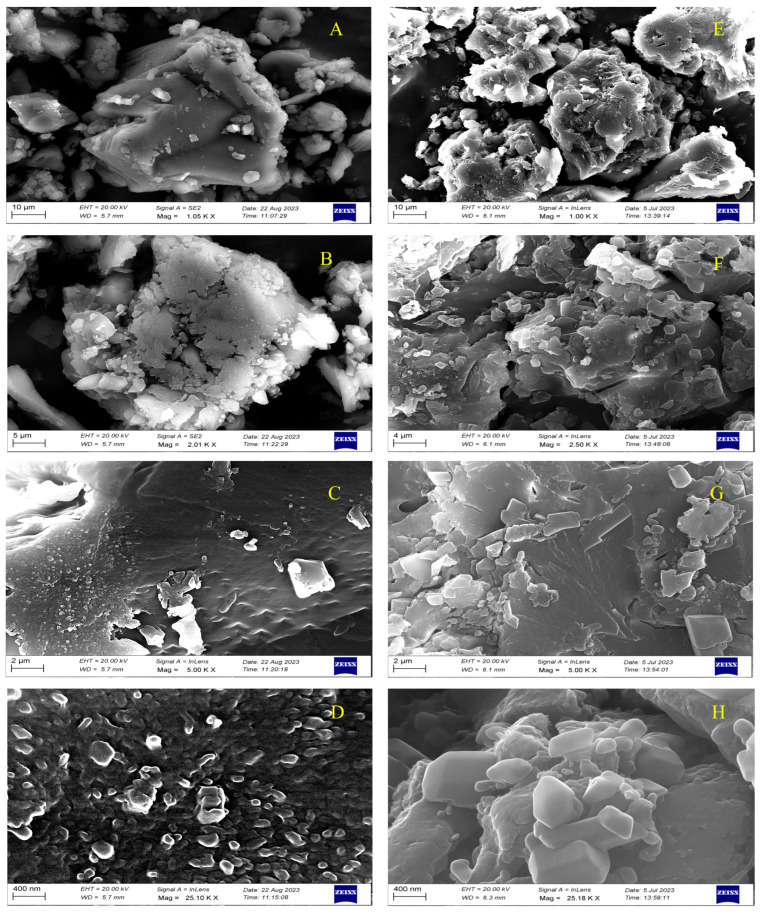
SEM micrographs of purified piperine crystals obtained via UBE (**A**–**D**) and UPE (**E**–**H**) at varying magnifications.

The surface morphology of piperine extracted using ultrasonic bath extraction (UBE) and ultrasonic probe extraction (UPE) was examined by SEM at varying magnifications (1.05 K–25.10 K×) using a ZEISS microscope operating at 20.00 kV with a working distance of 5.7 mm. UBE-extracted piperine exhibited well-defined crystalline morphology with discrete, equidimensional particles uniformly distributed across the field. At lower magnification (5 K×), the crystals displayed sharp edges and clear geometric boundaries, indicating preserved crystalline integrity. The surface appeared moderately rough with shallow grooves and occasional pores, suggesting that the mild cavitation generated during bath sonication effectively released piperine without inducing structural damage (Figure 8A–D) [54,66].

In contrast, UPE-derived piperine displayed a markedly different morphological profile characterized by extensive aggregation, irregular surface features, and pronounced peaks and valleys, reflecting intense mechanical disruption and localized cavitation effects. This morphological alteration is a direct consequence of the higher ultrasonic energy density and localized shear forces produced by the probe sonicator, which partially disrupt the crystalline lattice, increase surface roughness, and generate a larger specific surface area (Figure 8D,E) [67,68]. UPE-extracted samples appeared less uniform under SEM, this structural modification enhances solvent penetration, mass transfer, and the release of intracellular piperine. Therefore, the observed morphological distortion should be interpreted as a manifestation of effective ultrasonic energy transmission rather than a sign of degradation [22,67,69]. Overall, SEM analysis confirms that UBE preserves the native crystalline order of piperine, yielding morphologically stable particles, while UPE induces enhanced surface modification and microstructural disruption that contribute to superior extraction yield and bioactivity.

#### 3.6.10. EDS Elemental Analysis

**Figure 9:**
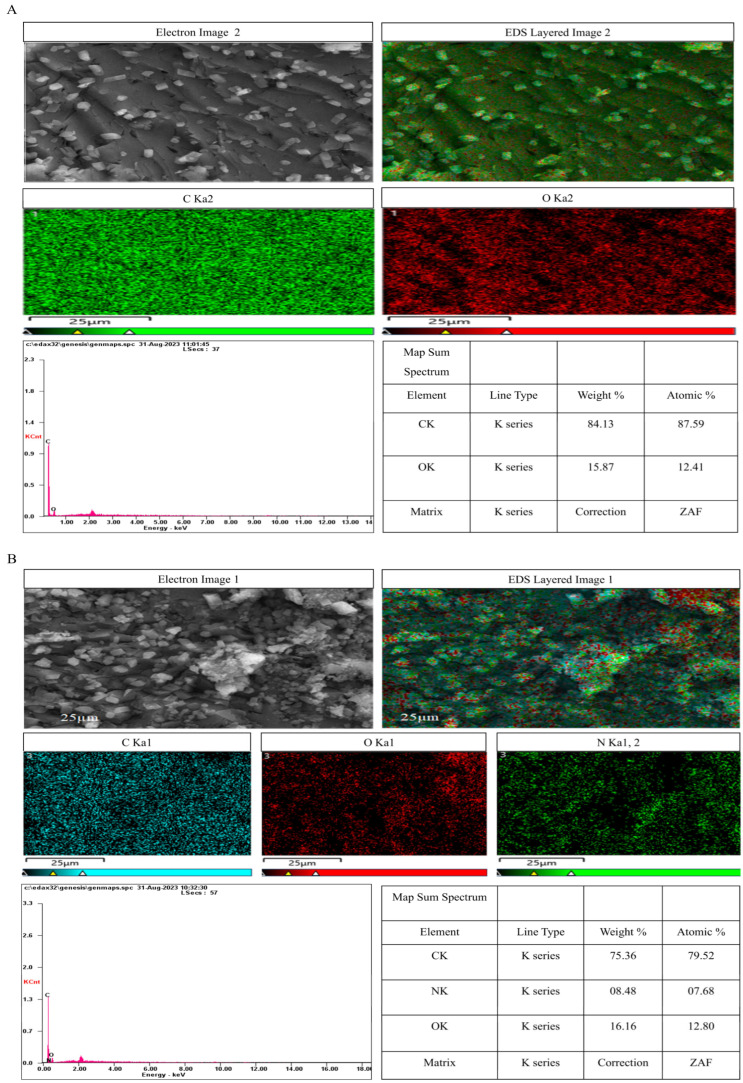
EDS spectra of piperine extracted by UBE (**A**) and UPE (**B**), highlighting differences in elemental composition and preservation of the amide functionality.

EDS analysis was used to semi-quantitatively analyze the elemental composition of UBE- and UPE-derived piperine crystals. Point analyses were performed at two distinct locations on each sample to assess elemental distribution uniformity. For UBE-piperine, the first point showed carbon (84.13 wt%) and oxygen (15.87 wt%) with nitrogen undetected, while the second point revealed carbon (50.21 wt%), nitrogen (2.55 wt%), and oxygen (8.11 wt%). This local variation suggests heterogeneous distribution or partial surface loss of nitrogen rather than complete degradation (Figure 9A; Supplementary Figure S4).

In contrast, UPE-piperine consistently displayed carbon (75.36 wt% and 77.92 wt%), nitrogen (8.48 wt% and 7.14 wt%), and oxygen (16.16 wt% and 14.94 wt%), aligning closely with the theoretical elemental composition of piperine (C_17_H_19_NO_3_) (Figure 9B; Supplementary Figure S5). The higher and more uniform nitrogen content in UPE samples supports better preservation of the amide functionality and improved sample homogeneity [38]. These observations complement the FTIR and Raman results, which confirmed the presence of amide and aromatic functional groups in both samples. Therefore, the lower nitrogen signal in UBE-piperine is likely due to localized variation or reduced surface detection sensitivity of EDS, rather than true chemical degradation of nitrogen-based groups.

#### 3.6.11. NMR Spectroscopy

**Figure 10:**
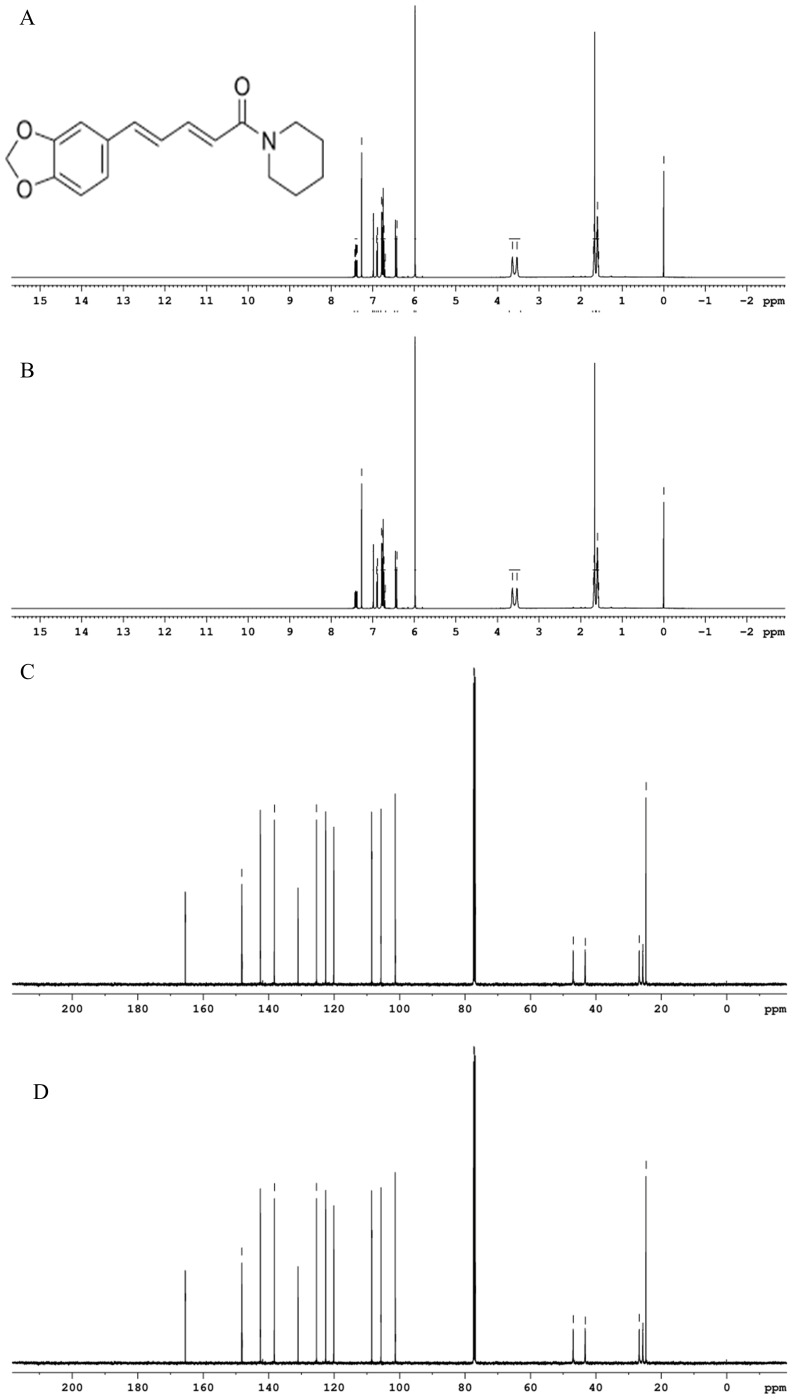
^1^H and ^13^C NMR spectra of purified piperine obtained from optimized UBE and UPE processes: (**A**) ^1^H NMR spectrum of UBE-piperine; (**B**) ^1^H NMR spectrum of UPE-piperine; (**C**) ^13^C NMR spectrum of UBE-piperine; and (**D**) ^13^C NMR spectrum of UPE-piperine, confirming identical chemical structures with improved signal resolution and purity in the UPE-derived sample.

^1^H and ^13^C NMR spectroscopy was performed to confirm the chemical identity and structural integrity of piperine obtained via UBE and UPE. The ^1^H NMR spectra of both UBE (Figure 10A) and UPE (Figure 10B) samples showed characteristic resonances at δ 1.60 (t, 3H, CH_3_–CH_2_), 2.52–2.58 (m, 2H, CH_2_–CH_2_), 3.90 (s, 3H, O–CH_3_), 5.92 (s, 2H, methylenedioxy –O–CH_2_–O–), and 6.45–7.65 (m, 7H, aromatic and olefinic protons), with identical chemical shifts, multiplicities, and coupling constants, confirming that the proton environment was unaffected by the extraction method. Minor differences in signal-to-noise ratios indicated slightly higher purity in the UPE sample, consistent with HPLC results. The ^13^C NMR spectra of UBE (Figure 10C) and UPE (Figure 10D) samples exhibited resonances at δ 14.2 (CH_3_), 27.8 (CH_2_), 56.1 (O–CH_3_), 101.4 (methylenedioxy –O–CH_2_–O–), 111.2–149.5 (aromatic C–C and C=C carbons), and 166.3 (amide carbonyl C=O), all in agreement with literature values for Piperine [70]. Notably, UPE-piperine displayed sharper and better-resolved carbonyl and aromatic signals, suggesting greater crystallinity and purity, which corroborates XRD and HPLC findings. Overall, the NMR data confirmed that both extraction methods preserved the full chemical structure of piperine, while UPE yielded a sample with marginally improved spectral resolution and purity.

### 3.7. In Vitro Evaluation

#### 3.7.1. Cytotoxicity of UBE- and UPE-Derived Piperine

Purified piperine obtained from UBE and UPE was evaluated for cytotoxic potential in C2C12 myoblast cells using the MTT assay. Both UBE- and UPE-derived piperine samples exhibited a concentration-dependent reduction in cell viability after 48 h of exposure. At lower concentrations (≤3.12 µg/mL), minimal cytotoxic effects were observed, with cell viability remaining above 85%. At higher doses (25–100 µg/mL), a marked decline in viability was evident, and UPE-derived piperine consistently showed greater inhibition of cell viability compared to UBE-derived piperine (Figure S6). The IC_50_ values were determined using the linear interpolation method, where the concentration corresponding to 50% inhibition was estimated by interpolating between two experimental points immediately above and below 50% cell viability. The calculated IC_50_ values based on this method were 40.6 µg/mL for UBE-piperine and 24.3 µg/mL for UPE-piperine, indicating relatively stronger cytotoxicity for the latter. Although both samples were tested at equivalent concentrations, the enhanced cytotoxic effect observed for UPE-piperine may be related not to extraction efficiency per se, but to ultrasonication-induced variations in physicochemical characteristics such as subtle differences in crystallinity, particle size, or solubility that can influence cellular uptake and bioavailability. Similar observations have been reported in earlier studies, where ultrasonic probe-assisted extraction yielded compounds with improved molecular dispersion and bioactivity compared to bath-assisted methods [22,71,72,73,74].

#### 3.7.2. Anticancer Activity Assessment

The anticancer activity of purified piperine obtained from UBE and UPE was evaluated in MCF-7 breast cancer and HT-29 colon cancer cells using the MTT assay (Figure S7). UPE-piperine consistently exhibited stronger cytotoxicity than UBE-piperine, particularly in MCF-7 cells where viability dropped to 39.36% at 15 µg/mL and 25.04% at 30 µg/mL, compared to 79.95% and 51.87% for UBE-piperine, while both showed comparable effects at 45 µg/mL. HT-29 cells were more sensitive, with UPE-piperine reducing viability to 62.34%, 20.96%, and 9.10% at 15, 30, and 45 µg/mL, respectively, compared to 88.52%, 41.87%, and 30.30% for UBE-Piperine. The IC_50_ for HT-29 cells was 19.47 µg/mL for UPE-piperine and 27.26 µg/mL for UBE-piperine, while IC_50_ values could not be reached in MCF-7 cells within the tested concentrations. Compared to the positive control doxorubicin, which yielded ~20–30% viability across both cell lines, UPE-piperine achieved comparable or superior efficacy in HT-29 cells at 45 µg/mL. These results highlight that extraction methodology significantly influences the bioactivity of piperine, with probe-assisted ultrasonication yielding samples of higher purity and structural integrity, thereby enhancing anticancer potency [4,54,72].

#### 3.7.3. Antioxidant Assays

**Figure 11:**
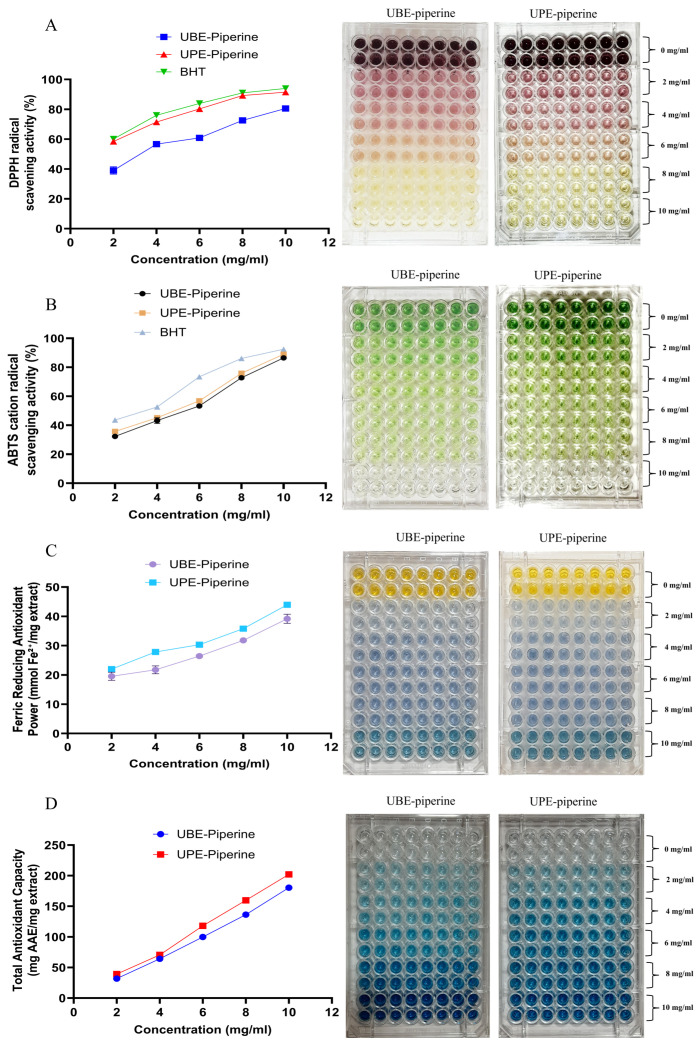
Antioxidant activity of purified piperine from UBE and UPE compared to the standard in 96-well plate assays, including (**A**) DPPH, (**B**) ABTS, (**C**) FRAP, and (**D**) TAC.

The antioxidant activity of purified piperine obtained from UBE and UPE was systematically evaluated using DPPH, ABTS, FRAP, and TAC assays at concentrations ranging from 2 to 10 mg/mL. In all assays, activity was concentration-dependent, and UPE-derived piperine consistently demonstrated superior radical scavenging and reducing potential compared to UBE-derived piperine. In the DPPH assay, UPE-piperine showed markedly higher free radical scavenging activity across all concentrations, reaching 91.50% inhibition at 10 mg/mL compared to 80.57% for UBE-piperine, closely approaching the standard BHT (93.96%). The radical scavenging was visually evident as a gradual fading of the deep purple DPPH solution to pale yellow, with UPE-piperine wells displaying a more pronounced color change at lower concentrations compared to UBE-piperine (note: color changes for the standard BHT are not shown; Figure 11A).

Similarly, in the ABTS assay, UPE-piperine exhibited enhanced activity, recording 89.01% inhibition at 10 mg/mL, whereas UBE-piperine showed 86.49%, approaching BHT (92.45%). The ABTS radical solution showed a color transition from intense green-blue to nearly colorless in UPE-piperine-treated wells, while UBE-piperine wells exhibited a slightly lighter green-blue at equivalent concentrations (Figure 11B).

The FRAP assay confirmed the higher reducing power of UPE-piperine, with an absorbance equivalent to 43.96 mmol Fe(II)/mg extract at 10 mg/mL compared to 39.17 mmol Fe(II)/mg for UBE-piperine. Wells containing UPE-piperine developed a more intense blue color, indicative of higher Fe(III) to Fe(II) reduction, compared to the relatively lighter blue observed in UBE-piperine wells (Figure 11C).

Consistent trends were observed in the TAC (phosphomolybdate) assay, where UPE-piperine demonstrated a strong total antioxidant capacity (202.10 ppm ascorbic acid equivalents at 10 mg/mL) compared to 180.33 ppm for UBE-piperine. The wells showed a progressive green-to-blue color intensification with increasing concentrations, with UPE-piperine wells consistently displaying darker hues than UBE-piperine, reflecting enhanced antioxidant capacity (Figure 11D).

Overall, the antioxidant assays collectively establish that UPE-derived piperine possesses significantly greater antioxidant potential than UBE-derived piperine, as evident both quantitatively and visually through color changes in the 96-well plates. This enhancement can be attributed to the superior extraction efficiency of ultrasonic probe technology, yielding piperine with higher purity, improved crystallinity, and possibly enhanced solubility, thereby increasing its radical scavenging and reducing capacity. These findings are consistent with previous reports of enhanced antioxidant activity in phytochemicals obtained via ultrasonic probe-assisted extraction [42,75,76,77].

#### 3.7.4. Intracellular ROS and RNS Evaluation in THP-1 and RAW 264.7 Cells

**Figure 12:**
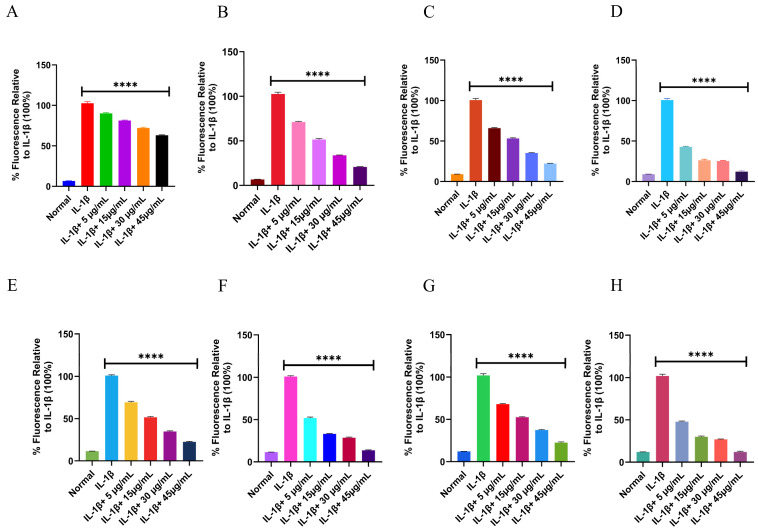
ROS and RNS levels in RAW 264.7 and THP-1 macrophages treated with purified piperine obtained from UBE and UPE. Panels (**A**) and (**B**) represent ROS inhibition in RAW 264.7 cells by UBE-piperine and UPE-piperine, respectively, while panels (**C**) and (**D**) show RNS inhibition in RAW 264.7 cells. Panels (**E**) and (**F**) depict ROS inhibition in THP-1 cells by UBE- and UPE-piperine, respectively, and panels (**G**) and (**H**) correspond to RNS inhibition in THP-1 cells. Data are presented as mean ± SD of triplicates. **** *p* < 0.001 was considered statistically significant compared to the control.

The intracellular antioxidant efficacy of piperine was evaluated in IL-1β-stimulated RAW 264.7 and THP-1 macrophages. In both cell lines, IL-1β treatment markedly elevated ROS and RNS levels compared to untreated controls. Treatment with piperine from UBE and UPE significantly reduced ROS and RNS levels in a dose-dependent manner (5–45 µg/mL). In RAW 264.7 cells, IL-1β increased ROS to ~100%, whereas co-treatment with UBE-piperine reduced ROS to 89.46%, 81.77%, 72.77%, and 62.53% at 5, 15, 30, and 45 µg/mL, respectively. UPE-piperine exerted a stronger suppression, reducing ROS to 70.23%, 51.00%, 33.31%, and 20.23% at corresponding concentrations. A similar trend was observed in RNS levels: UBE-piperine reduced RNS to 65.34%, 53.28%, 34.78%, and 21.91%, while UPE-piperine markedly decreased RNS to 42.82%, 27.54%, 25.13%, and 11.06% across increasing concentrations (Figure 12A–D).

In THP-1 cells, UBE-piperine reduced ROS to 69.42%, 51.53%, 34.68%, and 23.10% at 5, 15, 30, and 45 µg/mL, while UPE-piperine produced a stronger effect, lowering ROS to 52.53%, 32.58%, 28.37%, and 13.74%. Similarly, UBE-piperine decreased RNS to 67.67%, 52.00%, 36.56%, and 23.22%, whereas UPE-piperine further reduced RNS to 47.67%, 29.89%, 26.56%, and 12.33% at the same concentrations (Figure 12E–H). Collectively, these findings demonstrate that UPE-derived piperine exhibits a significantly greater capacity to suppress intracellular ROS and RNS compared to UBE-derived piperine in both macrophage models, confirming its superior antioxidant potential.

These intracellular results align with the chemical antioxidant assays, indicating that the enhanced suppression of ROS and RNS may stem from the higher purity and improved physicochemical properties of UPE-piperine, which likely promote greater cellular uptake and reactivity with intracellular oxidants. By attenuating both ROS and RNS, UPE-piperine provides stronger protection against oxidative and nitrosative stress, consistent with previous evidence that ultrasonic probe-assisted extraction enhances the bioactivity of phytochemicals [22,78,79,80,81].

## 4. Conclusions

This study demonstrated that ultrasonic probe extraction (UPE) using NADES is a more efficient strategy than ultrasonic bath extraction (UBE) for recovering piperine from *Piper nigrum* L. fruits. Response Surface Methodology (RSM) optimization confirmed a significantly higher piperine yield with UPE, attributable to enhanced cavitation intensity and improved mass transfer. The purified piperine obtained from both methods was comprehensively characterized by multiple analytical techniques, which verified its chemical identity, structural integrity, and high purity. Although the physicochemical characteristics of UBE- and UPE-derived piperine were largely comparable within the experimental error range, UPE consistently produced higher extraction efficiency and slightly improved bioactivity outcomes. Collectively, these results support that UPE offers a more reliable, rapid, and sustainable extraction method, yielding high-quality piperine suitable for nutraceutical, pharmaceutical, and functional food applications. Future studies may focus on scaling up the UPE-NADES process for industrial implementation, integrating it with continuous extraction systems, applying it to other bioactive alkaloids and spice-derived compounds, and coupling it with green purification or encapsulation strategies to enhance the stability and targeted delivery of piperine.

## Figures and Tables

**Table 1:** Composition of NADES used in ultrasound-assisted piperine extraction.

NADES Code	HBA	HBD	Molar Ratio	Type
NADES-1	L-Proline	Glycerin + Malic Acid	1:2:2	Ternary
NADES-2	Choline Chloride	Urea	1:1	Binary
NADES-3	Choline Chloride	1,2-Propylene Glycol	1:1	Binary
NADES-4	Choline Chloride	Malic Acid	1:1	Binary
NADES-5	Choline Chloride	Glycerin + Urea	1:1:1	Ternary
NADES-6	Choline Chloride	Citric Acid + 1,2-Propylene Glycol	1:2:2	Ternary

**Table 2:** Experimental levels of independent variables in UBE and UPE optimization study.

Independent Variable	Levels (UBE)					Levels (UPE)				
Liquid-to-Solid Ratio (mL/g)	10	15	20	25	30	15	20	25	30	35
Extraction Time (min)	15	30	45	60	75	20	35	50	65	80
Extraction Temperature (°C)	35	40	45	50	55	40	45	50	55	60
Water Content in NADES (%)	10	20	30	40	50	20	30	40	50	60

**Table 3:** BBD matrix for UBE-piperine optimization.

Levels	Liquid-to-Solid Ratio (mL/g)	Extraction Time (min)	Extraction Temperature (°C)	Water Content in NADES (%)
−1	20	30	40	10
0	25	45	45	20
1	30	60	50	30

**Table 4:** BBD matrix for UPE-piperine optimization.

Levels	Liquid-to-Solid Ratio (mL/g)	Extraction Time (min)	Extraction Temperature (°C)	Water Content in NADES (%)
−1	25	35	45	20
0	30	50	50	30
1	35	65	55	40

**Table 5:** Detailed experimental parameters for antioxidant assays.

Test	Chemicals and Experimental Setup	Reaction Time/Duration	Detection Wavelength (nm)	Control Standard	Data Analysis
DPPH	0.1 mM DPPH	35 min, dark	517 nm	BHT	% Radical Scavenging (Equation (4)) [44].
ABTS	7 mM ABTS + 2.45 mM K_2_S_2_O_8_	20 min, dark	734 nm	BHT	% Radical Scavenging (Equation (4)) [45].
FRAP	FRAP solution	15 min at 37 °C	593 nm	FeSO_4_	mmol Fe(II)/mg extract [46].
TAC	Phosphomolybdate reagent	95 min at 95 °C	695 nm	Ascorbic acid	ppm ascorbic acid equivalents [47].

**Table 6:** RSM Design Matrix with 29 Experimental Runs and Predicted Piperine Yield for UBE-Piperine Optimization.

Serial Number	A: Liquid-to-Solid Ratio (mL/g)	B: Extraction Time (min)	C: Extraction Temperature (°C)	D:Water Content in NADES (%)	Piperine Yield % (Predicted)
1	30	30	45	20	23.8
2	25	45	40	30	25.1
3	20	60	45	20	22.2
4	30	45	40	20	24.5
5	30	45	45	10	25.2
6	25	30	40	20	21.3
7	20	45	45	30	23.7
8	25	45	45	20	26.8
9	25	45	45	20	26.7
10	25	60	50	20	23.9
11	25	30	45	10	20.2
12	25	45	40	10	22.6
13	30	45	50	20	24.4
14	25	45	45	20	26.6
15	25	60	45	10	22.8
16	20	45	40	20	21.8
17	30	60	45	20	24.2
18	25	60	45	30	24.7
19	25	30	50	20	21.5
20	20	30	45	20	19.9
21	30	45	45	30	25.5
22	20	45	45	10	21.6
23	25	30	45	30	22.3
24	25	45	50	30	24.1
25	25	45	50	10	23.5
26	25	60	40	20	23.8
27	25	45	45	20	26.9
28	25	45	45	20	26.6
29	20	45	50	20	22.5

**Table 7:** RSM Design Matrix with 29 Experimental Runs and Predicted Piperine Yield for UPE-Piperine Optimization.

Serial Number	A: Liquid-to-Solid Ratio (mL/g)	B: Extraction Time (min)	C: Extraction Temperature (°C)	D: Water Content in NADES (%)	Piperine Yield % (Predicted)
1	35	65	50	30	44.6
2	30	35	45	30	38.7
3	25	65	50	30	41.7
4	30	50	45	40	44.3
5	25	50	55	30	43.1
6	30	35	50	40	41.5
7	35	50	50	40	47.4
8	30	50	55	40	47.6
9	30	65	45	30	43.2
10	30	50	50	30	49.6
11	30	35	50	20	38.5
12	25	50	50	40	41.9
13	25	50	50	20	36.7
14	30	65	55	30	46.2
15	30	50	45	20	39.5
16	35	50	45	30	42.7
17	30	50	55	20	42.3
18	30	50	50	30	49.9
19	35	50	50	20	44.1
20	25	35	50	30	37.8
21	30	50	50	30	50.1
22	25	50	45	30	38.8
23	30	65	50	20	41.2
24	35	50	55	30	45.8
25	35	35	50	30	41.1
26	30	65	50	40	45.7
27	30	50	50	30	49.2
28	30	35	55	30	42.1
29	30	50	50	30	49.5

## Data Availability

The data presented in this study are available in the article and Supplementary Materials.

## Supplementary Materials

The following supporting information can be downloaded at: https://www.mdpi.com/article/10.3390/biom15111631/s1. Supplementary Figure S1. Piperine yield obtained using different NADES formulations. Supplementary Figure S2. Contour maps corresponding to the response surface plots in Figure 4, illustrating the interactive effects of extraction parameters on piperine yield during UBE. Supplementary Figure S3. Contour maps corresponding to the response surface plots in Figure 5, illustrating the interactive effects of extraction parameters on piperine yield during UPE. Supplementary Figure S4. EDS spectra of piperine extracted by UBE recorded at two distinct surface points, highlighting variations in elemental composition and indicating partial surface loss or heterogeneous distribution of nitrogen while preserving the amide functionality. Supplementary Figure S5. EDS spectra of piperine extracted by UPE recorded at two distinct surface points, showing consistent elemental composition with uniform nitrogen distribution, confirming better preservation of the amide functionality and improved sample homogeneity. Supplementary Figure S6. Cytotoxicity assessment of UBE- and UPE-derived piperine in C2C12 cells. Supplementary Figure S7. Anticancer activity of UBE- and UPE-derived piperine against cancer cell lines. Supplementary Table S1. Analysis of variance (ANOVA) of the quadratic regression model for UBE-Piperine. Supplementary Table S2. Regression model summary statistics for UBE-Piperine. Supplementary Table S3. Analysis of variance (ANOVA) of the quadratic regression model for UPE-Piperine. Supplementary Table S4. Regression model summary statistics for UPE-Piperine.

## References

[1] Hashimoto K., Yaoi T., Koshiba H., Yoshida T., Maoka T., Fujiwara Y., Yamamoto Y., Mori K. (1996). Photochemical Isomerization of Piperine, a Pungent Constituent in Pepper. Food Sci. Technol. Int. Tokyo.

[2] Rahman Khan Z., Moni F., Sharmin S., Al-Mansur M.A., Gafur A., Rahman O., Afroz F. (2017). Isolation of Bulk Amount of Piperine as Active Pharmaceutical Ingredient (API) from Black Pepper and White Pepper (*Piper nigrum* L.). Pharmacol. Pharm..

[3] Shingate P.N., Dongre P.P., Kannur D.M. (2013). New method development for extraction and isolation of piperine from black pepper. Int. J. Pharm. Sci. Res..

[4] Tiwari A., Mahadik K.R., Gabhe S.Y. (2020). Piperine: A comprehensive review of methods of isolation, purification, and biological properties. Med. Drug Discov..

[5] Raman A., Lin Z. (2001). Use of Piperine for Treating Skin Pigmentation Disorders.

[6] Zhang C., Tian Q., Li Y. (2022). Design, synthesis, and insecticidal activity evaluation of piperine derivatives. Front. Chem..

[7] Vasavirama K., Upender M. (2014). Piperine: A valuable alkaloid from piper species. Int. J. Pharm. Pharm. Sci..

[8] Alshehri S., Imam S.S., Hussain A., Altamimi M.A. (2020). Formulation of piperine ternary inclusion complex using β CD and HPMC: Physicochemical characterization, molecular docking, and antimicrobial testing. Processes.

[9] Elnaggar Y.S.R., Etman S.M., Abdelmonsif D.A., Abdallah O.Y. (2015). Intranasal Piperine-Loaded Chitosan Nanoparticles as Brain-Targeted Therapy in Alzheimer’s Disease: Optimization, Biological Efficacy, and Potential Toxicity. J. Pharm. Sci..

[10] Arora S., Singh B., Kumar S., Kumar A., Singh A., Singh C. (2023). Piperine loaded drug delivery systems for improved biomedical applications: Current status and future directions. Health Sci. Rev..

[11] Yu J.W., Yuan H.W., Bao L.D., Si L.G. (2021). Interaction between piperine and genes associated with sciatica and its mechanism based on molecular docking technology and network pharmacology. Mol. Divers..

[12] Mitra S., Anand U., Jha N.K., Shekhawat M.S., Saha S.C., Nongdam P., Rengasamy K.R.R., Proćków J., Dey A. (2022). Anticancer Applications and Pharmacological Properties of Piperidine and Piperine: A Comprehensive Review on Molecular Mechanisms and Therapeutic Perspectives. Front. Pharmacol..

[13] Itharat A., Kanokkangsadal P., Khemawoot P., Wanichsetakul P., Davies N. (2020). Pharmacokinetics of piperine after oral administration of Sahastara remedy capsules in healthy volunteers. Res. Pharm. Sci..

[14] Tripathi A.K., Ray A.K., Mishra S.K. (2022). Molecular and pharmacological aspects of piperine as a potential molecule for disease prevention and management: Evidence from clinical trials. Beni-Suef Univ. J. Basic Appl. Sci..

[15] Poojar B., Ommurugan B., Adiga S., Thomas H., Sori R.K., Poojar B., Hodlur N., Tilak A., Korde R., Gandigawad P. (2017). Methodology Used in the Study. Asian J. Pharm. Clin. Res..

[16] Zhang Q.W., Lin L.G., Ye W.C. (2018). Techniques for extraction and isolation of natural products: A comprehensive review. Chin. Med..

[17] Pandit Patil S., Dasharath Lavate K., Bendgude R.R., MBhosale M., Ganpati S. (2023). Review: Conventional and Modern Extraction Methods of Herbal Drugs. Int. J. Pharm. Res. Appl..

[18] Sun S., Yu Y., Jo Y., Han J.H., Xue Y., Cho M., Bae S.-J., Ryu D., Park W., Ha K.-T. (2025). Impact of extraction techniques on phytochemical composition and bioactivity of natural product mixtures. Front. Pharmacol..

[19] Bitwell C., Indra S.S., Luke C., Kakoma M.K. (2023). A review of modern and conventional extraction techniques and their applications for extracting phytochemicals from plants. Sci. Afr..

[20] Cao S., Liang J., Chen M., Xu C., Wang X., Qiu L., Zhao X., Hu W. (2025). Comparative analysis of extraction technologies for plant extracts and absolutes. Front. Chem..

[21] Garcia-Larez F.L., Esquer J., Guzmán H., Zepeda-Quintana D.S., Moreno-Vásquez M.J., Rodríguez-Félix F., Del-Toro-Sánchez C.L., López-Corona B.E., Tapia-Hernández J.A. (2025). Effect of Ultrasound-Assisted Extraction (UAE) parameters on the recovery of polyphenols from pecan nutshell waste biomass and its antioxidant activity. Biomass Convers. Biorefinery.

[22] Shen L., Pang S., Zhong M., Sun Y., Qayum A., Liu Y., Rashid A., Xu B., Liang Q., Ma H. (2023). A comprehensive review of ultrasonic assisted extraction (UAE) for bioactive components: Principles, advantages, equipment, and combined technologies. Ultrason. Sonochem..

[23] Song Z., Huang G., Huang H. (2024). The ultrasonic-assisted enzymatic extraction, characteristics and antioxidant activities of lychee nuclear polysaccharide. Ultrason. Sonochem..

[24] Zorrilla J.G., Rial C., Martínez-González M.I., Molinillo J.M.G., Macías F.A., Varela R.M. (2024). Ginger Phytotoxicity: Potential Efficacy of Extracts, Metabolites and Derivatives for Weed Control. Agronomy.

[25] Shaterabadi D., Aboonajmi M., Ghorbani Javid M., Arabhosseini A. (2020). Effect of power ultrasound on the extraction of black caraway (*Carum carvi* L.) and evaluation of their qualitative properties using response surface methodology. Food Sci. Nutr..

[26] Sganzerla M., Coutinho J.P., de Melo A.M.T., Godoy H.T. (2014). Fast method for capsaicinoids analysis from Capsicum chinense fruits. Food Res. Int..

[27] Freitas D.S., Ribeiro A., Cavaco-paulo A. (2025). The Versatility of NADES Across Applications. Molecules.

[28] Kocanci F.G., Dolanbay S.N., Aslim B. (2022). Comparison of three different protocols of alkaloid extraction from Glaucium corniculatum plant. Int. J. Second. Metab..

[29] Masala V., Jokić S., Aladić K., Molnar M., Tuberoso C.I.G. (2024). Exploring Phenolic Compounds Extraction from Saffron Water Extraction. Molecules.

[30] Lwamba C., Aboushanab S.A., Ambati R.R., Kovaleva E.G. (2023). Innovative Green Approach for Extraction of Piperine from Black Pepper Based on Response Surface Methodology. Sustain. Chem..

[31] Stasiłowicz-Krzemień A., Wójcik J., Gościniak A., Szymański M., Szulc P., Górecki K., Cielecka-Piontek J. (2024). Natural Deep Eutectic Solvents Combined with Supercritical Carbon Dioxide for the Extraction of Curcuminoids from Turmeric. Pharmaceuticals.

[32] Chevé-Kools E., Choi Y.H., Roullier C., Ruprich-Robert G., Grougnet R., Chapeland-Leclerc F., Hollmann F. (2025). Natural deep eutectic solvents (NaDES): Green solvents for pharmaceutical applications and beyond. Green Chem..

[33] Jiménez-Ortega L.A., Bastidas-Bastidas P.d.J., Angulo-Escalante M.A., Mota-Morales J.D., Heredia J.B. (2024). Optimized Extraction of Glycosylated Flavonoids from Pepper (*Capsicum annuum* L.) Agricultural Biomass Residues through Ultrasonic Pulse-Assisted Natural Deep Eutectic Solvents. ACS Sustain. Resour. Manag..

[34] Wu K., Ren J., Wang Q., Nuerjiang M., Xia X., Bian C. (2022). Research Progress on the Preparation and Action Mechanism of Natural Deep Eutectic Solvents and Their Application in Food. Foods.

[35] Ferreira C., Sarraguça M. (2024). A Comprehensive Review on Deep Eutectic Solvents and Its Use to Extract Bioactive Compounds of Pharmaceutical Interest. Pharmaceuticals.

[36] Aguilar-Hernández G., Zepeda-Vallejo L.G., García-Magaña M.D.L., Vivar-Vera M.D.L.Á., Pérez-Larios A., Girón-Pérez M.I., Coria-Tellez A.V., Rodríguez-Aguayo C., Montalvo-González E. (2020). Extraction of alkaloids using ultrasound from pulp and by-products of soursop fruit (*Annona muricata* L.). Appl. Sci..

[37] Kazmi I., Al-Abbasi F.A., Imam S.S., Afzal M., Nadeem M.S., Altayb H.N., Alshehri S. (2022). Formulation of Piperine Nanoparticles: In Vitro Breast Cancer Cell Line and In Vivo Evaluation. Polymers.

[38] Latiff N.A., Ong P.Y., Abd Rashid S.N.A., Abdullah L.C., Mohd Amin N.A., Fauzi N.A.M. (2021). Enhancing recovery of bioactive compounds from Cosmos caudatus leaves via ultrasonic extraction. Sci. Rep..

[39] Tang Z., Wang Y., Huang G., Huang H. (2023). Ultrasound-assisted extraction, analysis and antioxidant activity of polysaccharide from the rinds of *Garcinia mangostana* L.. Ultrason. Sonochem..

[40] Grozdanova T., Trusheva B., Alipieva K., Popova M., Dimitrova L., Najdenski H., Zaharieva M.M., Ilieva Y., Vasileva B., Miloshev G. (2020). Extracts of medicinal plants with natural deep eutectic solvents: Enhanced antimicrobial activity and low genotoxicity. BMC Chem..

[41] Jeliński T., Przybyłek M., Cysewski P. (2019). Natural Deep Eutectic Solvents as Agents for Improving Solubility, Stability and Delivery of Curcumin. Pharm. Res..

[42] Deng L., Huang G. (2025). Ultrasound-assisted extraction, optimization, characteristics and antioxidant activity of *Piper nigrum* L. polysaccharides. Ultrason. Sonochem..

[43] Paarakh P.M., Sreeram D.C., Shruthi S.D., Ganapathy S.P. (2015). In vitro cytotoxic and in silico activity of piperine isolated from *Piper nigrum* fruits Linn. Silico Pharmacol..

[44] Riley R., Chapman V. (1958). Genetic control of the cytologically diploid behaviour of hexaploid wheat. Nature.

[45] Stämpfli R., Brühwiler P., Mourad S., Verdejo R., Shaffer M. (2007). Development and characterisation of carbon nanotube-reinforced polyurethane foams. EMPA Act..

[46] Salari S., Bahabadi S.E., Samzadeh-Kermani A., Yosefzaei F. (2019). In-vitro evaluation of antioxidant and antibacterial potential of green synthesized silver nanoparticles using prosopis farcta fruit extract. Iran. J. Pharm. Res..

[47] Das P.E., Abu-Yousef I.A., Majdalawieh A.F., Narasimhan S., Poltronieri P. (2020). Green Synthesis of Encapsulated Copper Nanoparticles Using a Hydroalcoholic Extract of Moringa oleifera Leaves and Assessment of Their Antioxidant and Antimicrobial Activities. Molecules.

[48] Zhong G., Yang X., Jiang X., Kumar A., Long H., Xie J., Zheng L., Zhao J. (2019). Dopamine-melanin nanoparticles scavenge reactive oxygen and nitrogen species and activate autophagy for osteoarthritis therapy. Nanoscale.

[49] Bencresciuto G.F., Carnevale M., Paris E., Gallucci F., Santangelo E., Migliori C.A. (2025). A Sustainable Alternative for Cosmetic Applications: NADES Extraction of Bioactive Compounds from Hazelnut By-Products. Sustainability.

[50] García-Roldán A., Piriou L., Jauregi P. (2023). Natural deep eutectic solvents as a green extraction of polyphenols from spent coffee ground with enhanced bioactivities. Front. Plant Sci..

[51] Spaggiari C., Carbonell-Rozas L., Zuilhof H., Costantino G., Righetti L. (2025). Structural elucidation and long-term stability of synthesized NADES: A detailed physicochemical analysis. J. Mol. Liq..

[52] Maimulyanti A., Prihadi A.R., Mellisani B., Nurhidayati I., Putri F.A.R., Puspita F., Widarsih R.W. (2023). Green Extraction Technique To Separate Tannin From Coffee Husk Waste Using Natural Deep Eutectic Solvent (Nades). Rasayan J. Chem..

[53] Putu N., Hikmawanti E., Ramadon D., Jantan I. (2021). Natural Deep Eutectic Solvents (NADES): Phytochemical Extraction Performance Enhancer for Pharmaceutical and Nutraceutical Product Development. Plants.

[54] Ramos I.N.d.F., da Silva M.F., Lopes J.M.S., Cruz J.N., Alves F.S., do Rego J.d.A.R., Costa M.L.d., Assumpção P.P.d., Barros Brasil D.d.S., Khayat A.S. (2023). Extraction, Characterization, and Evaluation of the Cytotoxic Activity of Piperine in Its Isolated form and in Combination with Chemotherapeutics against Gastric Cancer. Molecules.

[55] Chumanee S., Thunta J., Khoomsab R., Winyakul C. (2024). Optimizing ultrasound-assisted extraction for enhanced quantification of 7-hydroxymitragynine and mitragynine in kratom. Creat. Sci..

[56] Yohannes A., Zhang B., Dong B., Yao S. (2019). Ultrasonic extraction of tropane alkaloids from radix physochlainae using as extractant an ionic liquid with similar structure. Molecules.

[57] Jauregi P., Esnal-Yeregi L., Labidi J. (2024). Natural deep eutectic solvents (NADES) for the extraction of bioactives: Emerging opportunities in biorefinery applications. PeerJ Anal. Chem..

[58] Nguyen K.V., Le N.T., Dang V.T.T., Koshovyi O., Raal A., Nguyen H.T. (2025). Alkaloid Extraction from Coptis chinensis Franch. Using Ultrasound-Assisted Aqueous Solutions of Surfactants, Organic Acids, Deep Eutectic Solvents, and Supramolecular Deep Eutectic Solvents. Molecules.

[59] Le X.T., Nguyen N.T., Nguyen-Thi B.T., Dang M.K., Ngo-Thi T.N., Vu D.P., Duong-Nguyen H.N., Pham-Vu M.C. (2024). Optimization of Piperine Extraction Process from Vietnamese White Pepper. IOP Conf. Ser. Earth Environ. Sci..

[60] Singh Kumar N., Kumar P.K., Gupta Kumar D., Singh S., Kumar Singh V. (2011). Scholars Research Library UV-spectrophotometric method development for estimation of piperine in Chitrakadi Vati. Sch. Res. Libr..

[61] Al-Mamun M.R., Maniruzzaman M., Rahman Badal M.M., Haque M.A. (2024). Comparison of piperine content, antimicrobial and antioxidant activity of Piper chaba root and stem. Heliyon.

[62] Han Jeong Y., Van Kien N., Jin Han Seog D., Ryoo J.J. (2022). Comparison between the use of polyether ether ketone and stainless steel columns for ultrasonic-assisted extraction under various ultrasonic conditions. Ultrason. Sonochem..

[63] Rathod S.S., Rathod V.K. (2014). Extraction of piperine from Piper longum using ultrasound. Ind. Crops Prod..

[64] Kumar K., Srivastav S., Sharanagat V.S. (2021). Ultrasound assisted extraction (UAE) of bioactive compounds from fruit and vegetable processing by-products: A review. Ultrason. Sonochem..

[65] Alves F.S., Rodrigues Do Rego J.d.A., Da Costa M.L., Lobato Da Silva L.F., Da Costa R.A., Cruz J.N., Brasil D.D.S.B. (2020). Spectroscopic methods and in silico analyses using density functional theory to characterize and identify piperine alkaloid crystals isolated from pepper (*Piper nigrum* L.). J. Biomol. Struct. Dyn..

[66] Imam S.S., Alzahrani T.A., Hussain A., Altamimi M.A. (2020). Formulation and Evaluation of Supramolecular Food-Grade Piperine HP β CD and TPGS Complex: Dissolution, Physicochemical Characterization, Molecular Docking, In Vitro Antioxidant Activity, and Antimicrobial Assessment. Molecules.

[67] Zhao S., Kwok K.C., Liang H. (2007). Investigation on ultrasound assisted extraction of saikosaponins from Radix Bupleuri. Sep. Purif. Technol..

[68] Bucur M.P., Radulescu M.C., Radu G.L., Bucur B. (2023). Cavitation-Effect-Based Treatments and Extractions for Superior Fruit and Milk Valorisation. Molecules.

[69] Tan S.X., Andriyana A., Lim S., Ong H.C., Pang Y.L., Ngoh G.C. (2021). Rapid Ultrasound-Assisted Starch Extraction from Sago Pith Waste (SPW) for the Fabrication of Sustainable Bioplastic Film. Polymers.

[70] Mohanraj V., Aravindan B., Jayaprakash C., Thenmozhi M. (2014). In Silico Drug Design and Extraction of Piperine an inhibitor for fernesyltransferase in *Cryptococcus neoformans*. World J. Pharm. Res..

[71] Haiss M.A., Maraie N.K. (2021). Utilization of ultrasonication technique for the preparation of apigenin nanocrystals. Int. J. Drug Deliv. Technol..

[72] Naik A.S., Suryawanshi D., Kumar M., Waghmare R. (2021). Ultrasonic treatment: A cohort review on bioactive compounds, allergens and physico-chemical properties of food. Curr. Res. Food Sci..

[73] Chemat F., Rombaut N., Sicaire A.G., Meullemiestre A., Fabiano-Tixier A.S., Abert-Vian M. (2017). Ultrasound assisted extraction of food and natural products. Mechanisms, techniques, combinations, protocols and applications. A review. Ultrason. Sonochem..

[74] McDonnell C., Tiwari B.K. (2017). Ultrasound: A Clean, Green Extraction Technology for Bioactives and Contaminants.

[75] Mehta N., S J., Kumar P., Verma A.K., Umaraw P., Khatkar S.K., Khatkar A.B., Pathak D., Kaka U., Sazili A.Q. (2022). Extracción asistida por ultrasonidos y encapsulación de componentes bioactivos para aplicaciones alimentarias. Foods.

[76] Giri S., Dash K.K., Bhagya Raj G.V.S., Kovács B., Ayaz Mukarram S. (2024). Ultrasound assisted phytochemical extraction of persimmon fruit peel: Integrating ANN modeling and genetic algorithm optimization. Ultrason. Sonochem..

[77] Cho S., Jung Y., Rho S.J., Kim Y.R. (2025). Stability, bioavailability, and cellular antioxidant activity of piperine complexed with cyclic glucans. Food Sci. Biotechnol..

[78] Chang W.L., Peng J.Y., Hong C.L., Li P.C., Chye S.M., Lu F.J., Lin H.Y., Chen C.H. (2025). Piperine Induces Apoptosis and Cell Cycle Arrest via Multiple Oxidative Stress Mechanisms and Regulation of PI3K/Akt and MAPK Signaling in Colorectal Cancer Cells. Antioxidants.

[79] Uniyal P., Akhtar A., Rawat R. (2025). Flavonoid-Based Combination Therapies and Nano-Formulations: An Emerging Frontier in Breast Cancer Treatment. Pharmaceuticals.

[80] Wen C., Zhang J., Zhang H., Dzah C.S., Zandile M., Duan Y., Ma H., Luo X. (2018). Advances in ultrasound assisted extraction of bioactive compounds from cash crops—A review. Ultrason. Sonochem..

[81] Bin Mokaizh A.A., Nour A.H., Kerboua K. (2024). Ultrasonic-assisted extraction to enhance the recovery of bioactive phenolic compounds from Commiphora gileadensis leaves. Ultrason. Sonochem..

