# Supplementary material for: Comparative Ultrasonic Bath and Probe Extraction of Piperine from Piper nigrum L. Using Natural Deep Eutectic Solvents: RSM Optimization, Characterization, and In Vitro Bioactivity
Source: Biomolecules. 2025 Nov 20;15(11):1631. doi: 10.3390/biom15111631 (PMC12650695; doi:10.3390/biom15111631)
Supplement: Supplementary file 1 [file biomolecules-15-01631-s001.zip › biomolecules-3943279 - Supplementary files-pdf/Supplementary_Figures_Piperine.pdf]

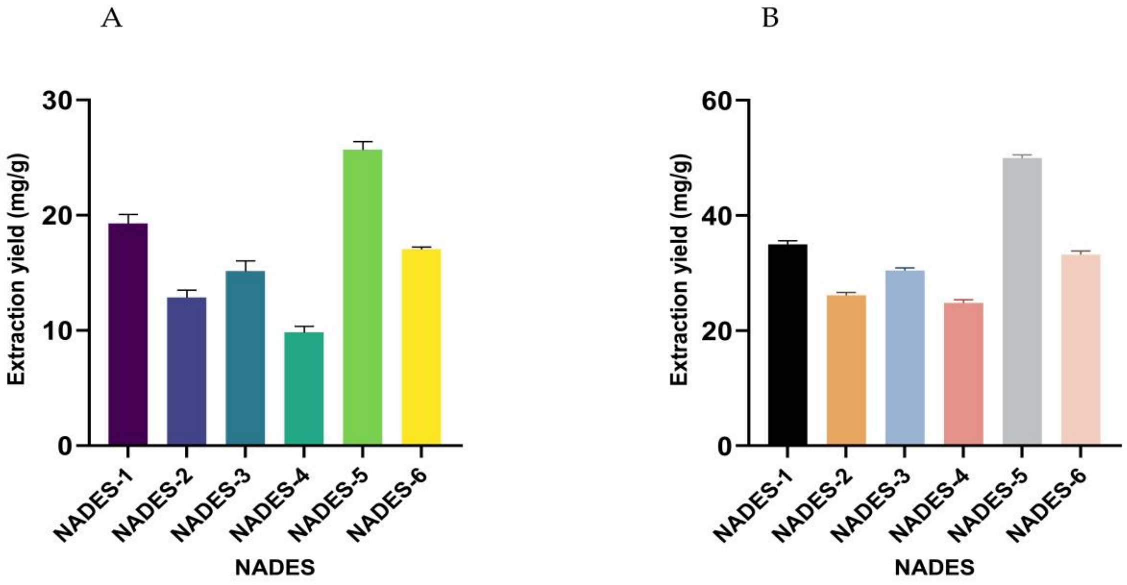

Supplementary Figure S1. Piperine yield obtained using different NADES formulations: **(A)** UBE. **(B)** UPE.

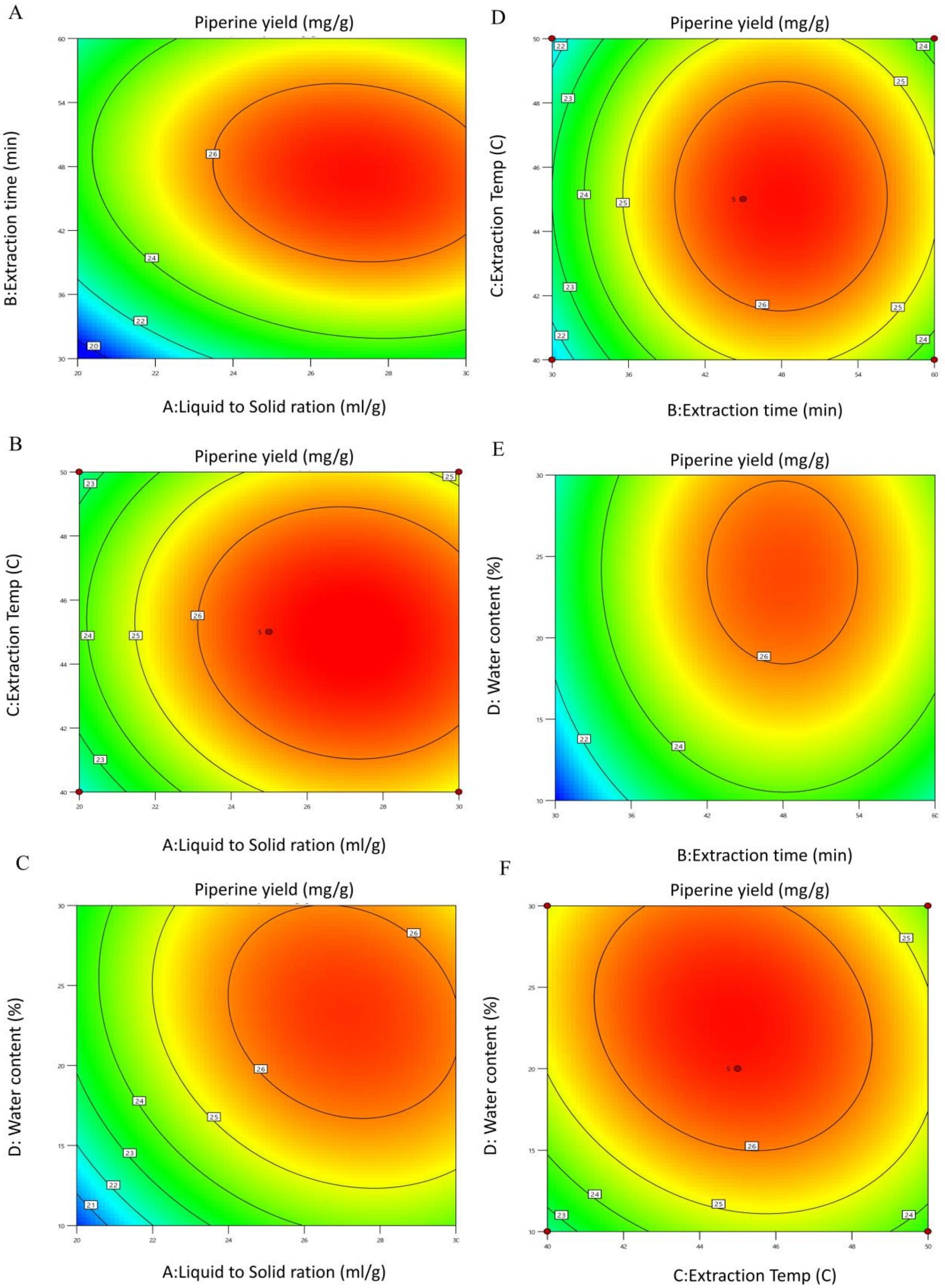
A
Piperine yield (mg/g)
B:Extraction time (min)
A:Liquid to Solid ration (ml/g)
D
Piperine yield (mg/g)
C:Extraction Temp (C)
B:Extraction time (min)
B
Piperine yield (mg/g)
C:Extraction Temp (C)
A:Liquid to Solid ration (ml/g)
E
Piperine yield (mg/g)
D: Water content (%)
B:Extraction time (min)
C
Piperine yield (mg/g)
D: Water content (%)
A:Liquid to Solid ration (ml/g)
F
Piperine yield (mg/g)
D: Water content (%)
C:Extraction Temp (C)

Supplementary Figure S2. Contour maps corresponding to the response surface plots in Figure 4, illustrating the interactive effects of extraction parameters on piperine yield during UBE. Interactions include: (**A**) liquid-to-solid ratio × extraction time, (**B**) liquid-to-solid ratio × temperature, (**C**) liquid-to-solid ratio × water content, (**D**) extraction time × temperature, (**E**) extraction time × water content, and (**F**) temperature × water content.

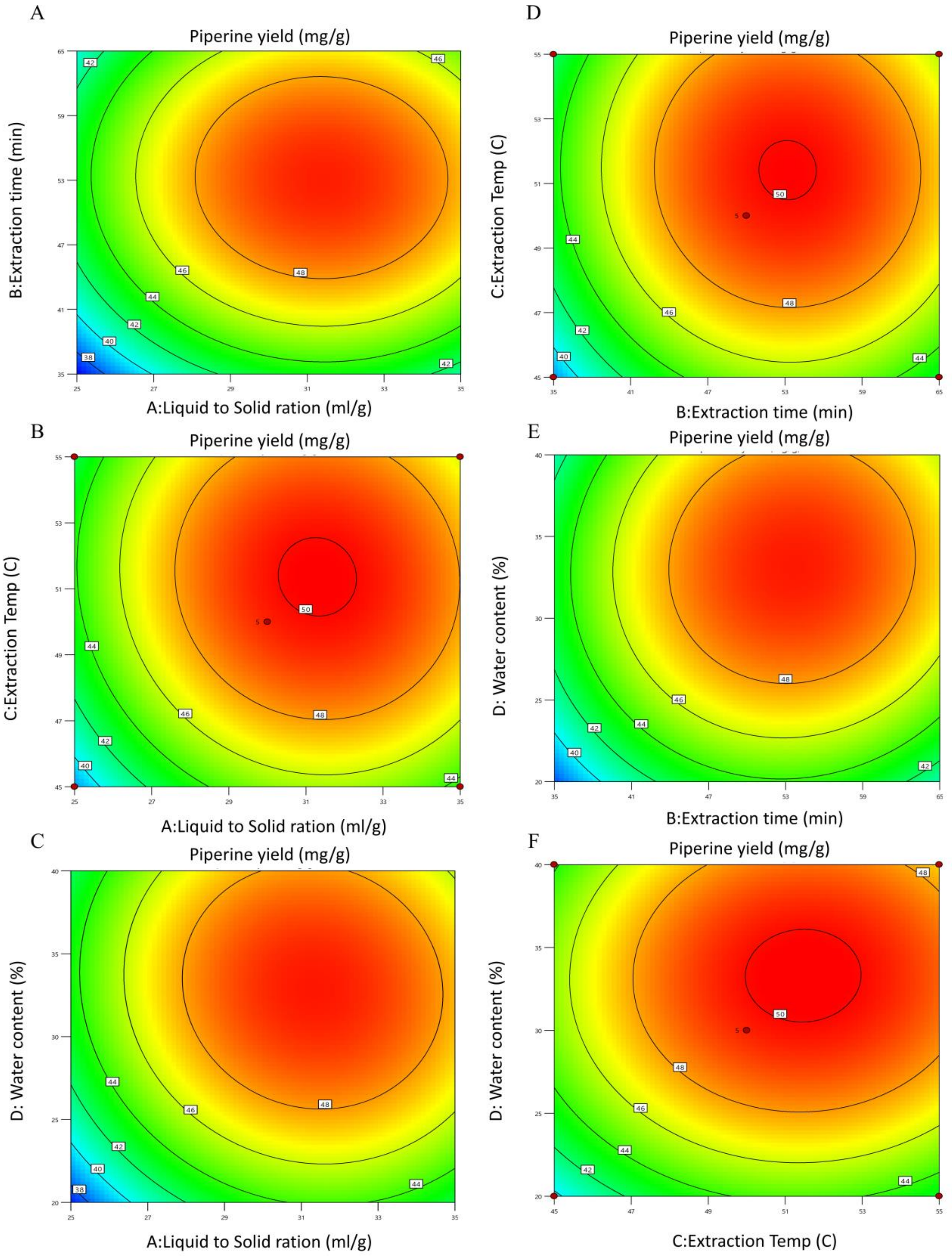

Supplementary Figure S3. Contour maps corresponding to the response surface plots in Figure 5, illustrating the interactive effects of extraction parameters on piperine yield during UPE. Interactions include: (**A**) liquid-to-solid ratio

× extraction time, (**B**) liquid-to-solid ratio × temperature, (**C**) liquid-to-solid ratio × water content, (**D**) extraction time × temperature, (**E**) extraction time × water content, and (**F**) temperature × water content.

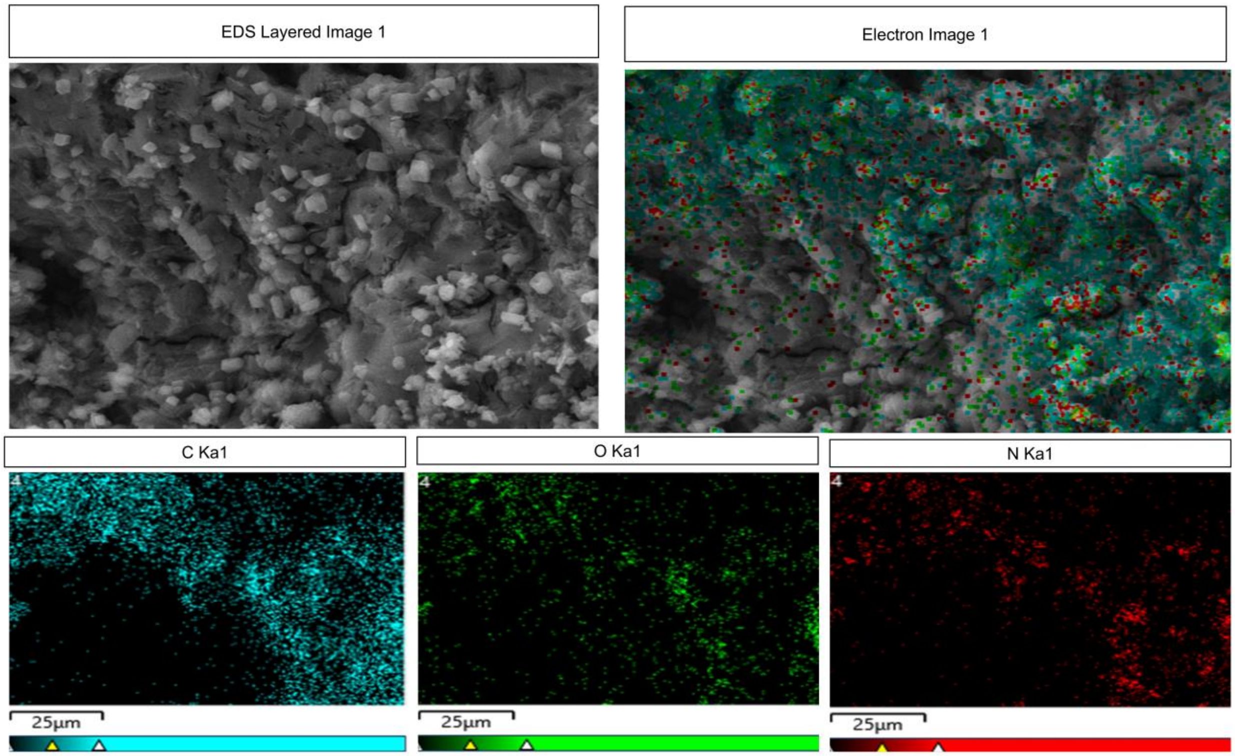

Map Sum Spectrum			
Element	Line Type	Weight %	Atomic %
CK	K series	50.21	59.23
NK	K series	02.55	03.42
OK	K series	08.11	7.21
Matrix	K series	Correction	ZAF

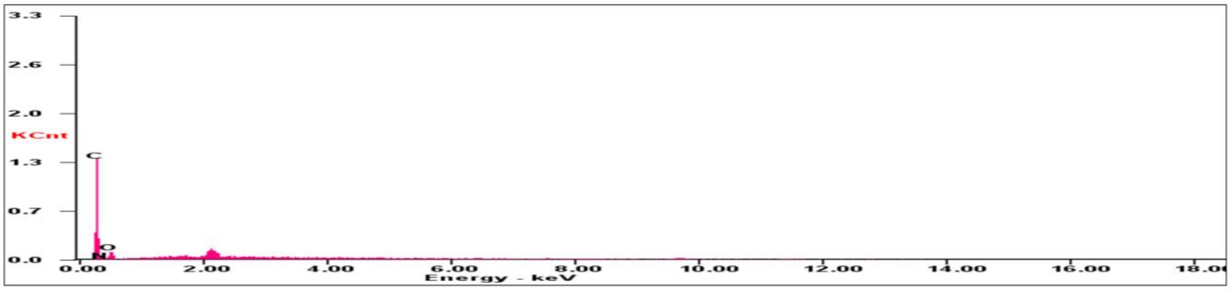

Supplementary Figure S4. EDS spectra of piperine extracted by UBE recorded at two distinct surface points, highlighting variations in elemental composition and indicating partial surface loss or heterogeneous distribution of nitrogen while preserving the amide functionality.

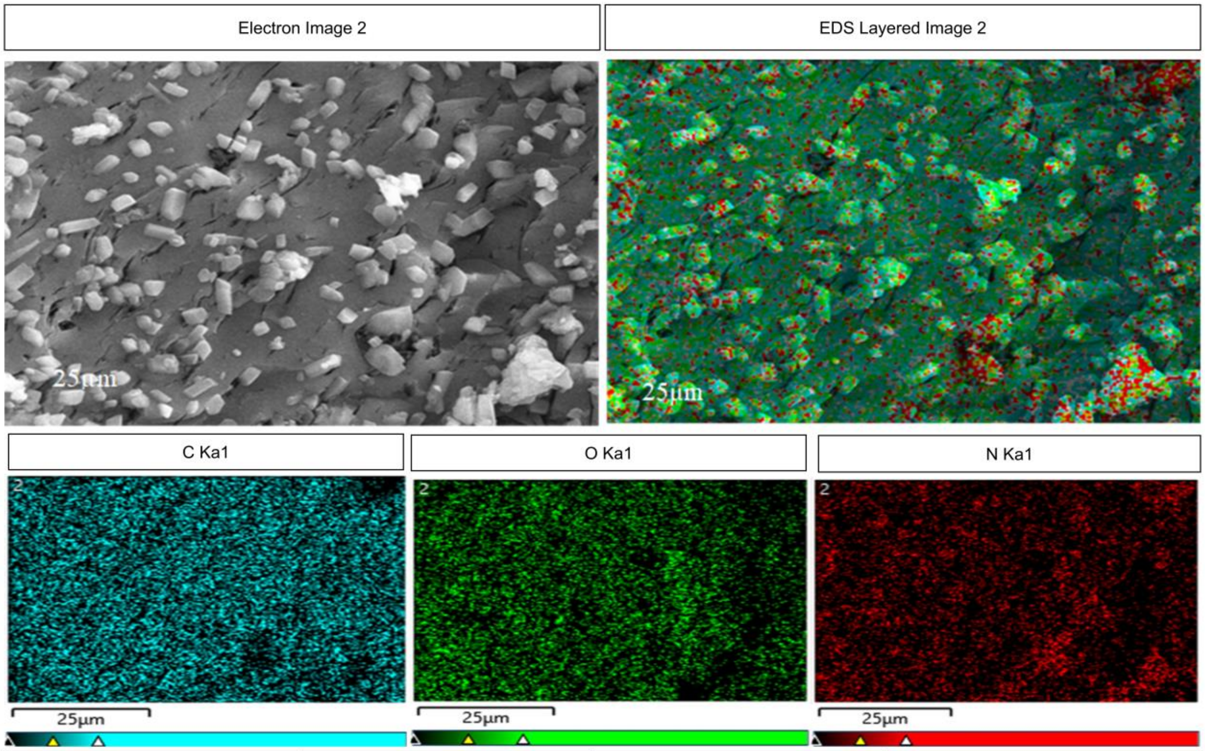

Map Sum Spectrum			
Element	Line Type	Weight %	Atomic %
CK	K series	77.92	81.80
NK	K series	07.14	06.43
OK	K series	14.94	11.77
Matrix	K series	Correction	ZAF

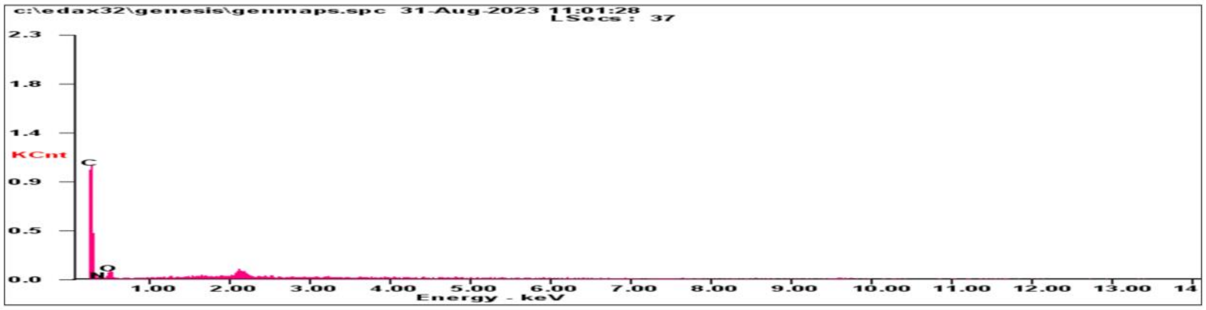

Supplementary Figure S5. EDS spectra of piperine extracted by UPE recorded at two distinct surface points, showing consistent elemental composition with uniform nitrogen distribution, confirming better preservation of the amide functionality and improved sample homogeneity.

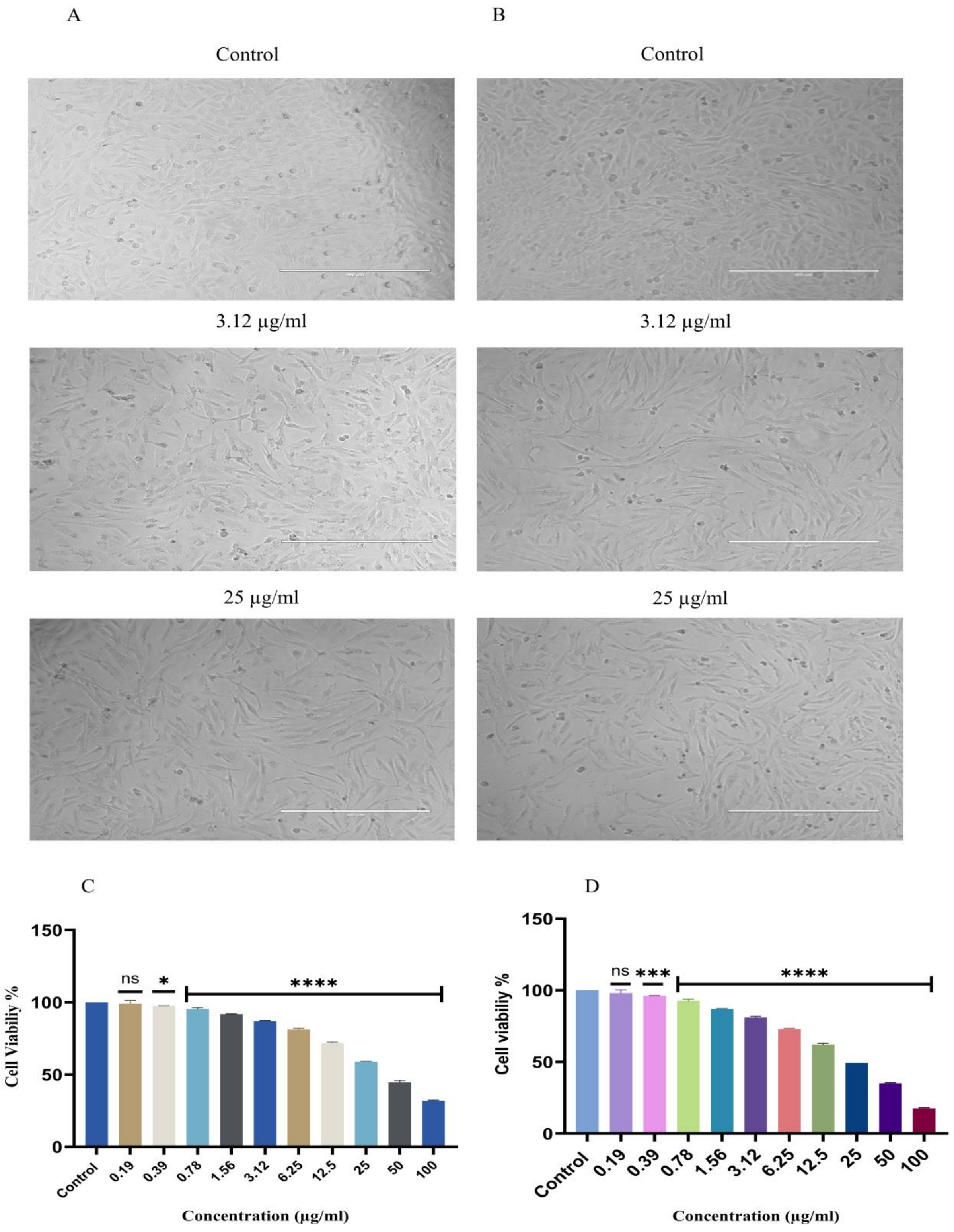

Supplementary Figure S6. Cytotoxicity assessment of UBE- and UPE-derived piperine in C2C12 cells: (**A**) UBE-treated at 400× magnification, (**B**) UPE-treated at 400× magnification, (**C**) Cell viability (%) for UBE, and (**D**) for UPE.

A- MCF-7

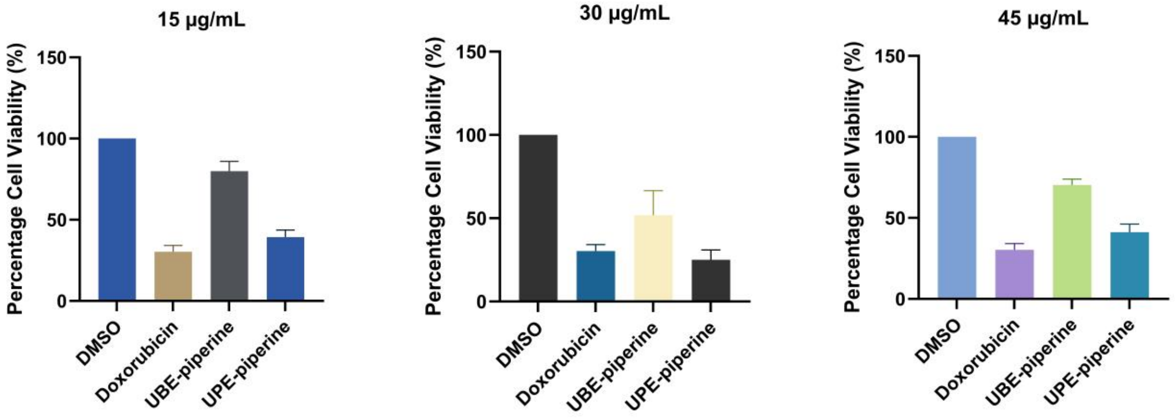

B-TH-29

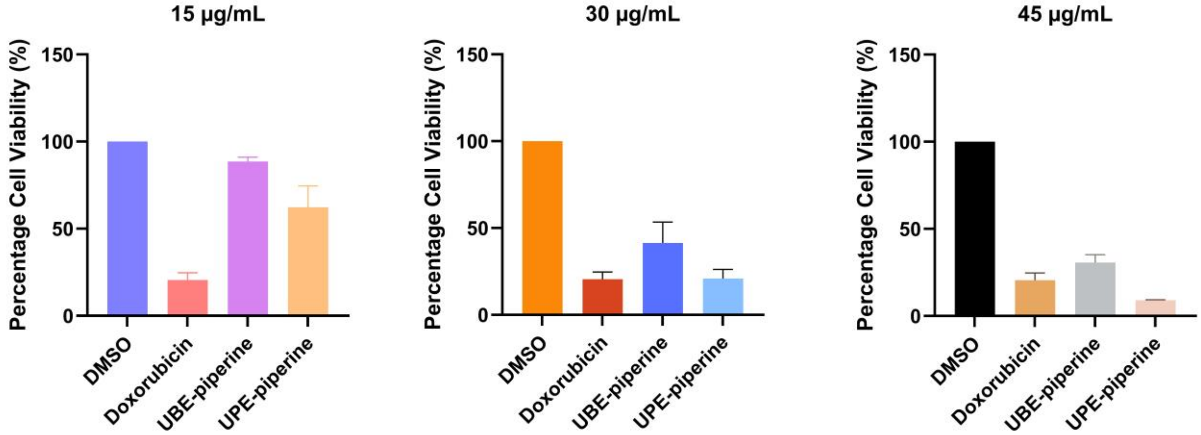

Supplementary Figure S7. Anticancer activity of UBE- and UPE-derived piperine against cancer cell lines: (**A**) Cell viability of MCF-7 cells, (**B**) Cell viability of HT-29 cells.
