# Supplementary material for: Comparative Ultrasonic Bath and Probe Extraction of Piperine from Piper nigrum L. Using Natural Deep Eutectic Solvents: RSM Optimization, Characterization, and In Vitro Bioactivity
Source: Biomolecules. 2025 Nov 20;15(11):1631. doi: 10.3390/biom15111631 (PMC12650695; doi:10.3390/biom15111631)
Supplement: Supplementary file 1 [file biomolecules-15-01631-s001.zip › biomolecules-3943279 - Supplementary files-pdf/Supplementary_Tables_Piperine .pdf]

Supplementary Table S1. Analysis of variance (ANOVA) of the quadratic regression model for UBE-Piperine.

Source (term)	Sum of Squares	df	Mean Square	F-value	p-value	Significance
Model	195.1656	14	13.9404	88.0238	<0.0001	**
A: Solid–liquid ratio	18.8468	1	18.8468	119.0054	<0.0001	**
B: Extraction time	51.5132	1	51.5132	325.2734	<0.0001	**
C: Temperature	16.9501	1	16.9501	107.0293	<0.0001	**
D: Water content	14.5842	1	14.5842	92.0899	<0.0001	**
A^2	16.1246	1	16.1246	101.8167	<0.0001	**
B^2	46.0397	1	46.0397	290.7114	<0.0001	**
C^2	16.9008	1	16.9008	106.7176	<0.0001	**
D^2	11.4165	1	11.4165	72.0880	<0.0001	**
A × B	0.9025	1	0.9025	5.6987	0.0316	*
A × C	0.1600	1	0.1600	1.0103	0.3319	n.s.
A × D	0.8100	1	0.8100	5.1146	0.0402	*
B × C	0.0025	1	0.0025	0.0158	0.9018	n.s.
B × D	0.0100	1	0.0100	0.0631	0.8053	n.s.
C × D	0.9025	1	0.9025	5.6987	0.0316	*
Residual	2.2172	14	0.1584	–	–	–
Correlation total	197.3828	28	–	–	–	–

Annotation: ** $p < 0.01$ (highly significant); * $p < 0.05$ (significant); n.s. = not significant.

Supplementary Table S2. Regression model summary statistics for UBE-Piperine.

Statistic	Value
Std. Dev.	0.398
Mean (Yield)	23.748
C.V. %	1.676
PRESS	12.485
R^2	0.9796
Adjusted R^2	0.9592
Predicted R^2	0.8851
Adeq Precision	17.82

Supplementary Table S3. Analysis of variance (ANOVA) of the quadratic regression model for UPE-Piperine.

Source (term)	Sum of Squares	df	Mean Square	F-value	p-value	Significance
Model	201.342	14	14.3816	95.213	<0.0001	**
A: Solid–liquid ratio	20.125	1	20.125	133.03	<0.0001	**
B: Extraction time	52.845	1	52.845	365.63	<0.0001	**
C: Temperature	18.442	1	18.442	127.61	<0.0001	**
D: Water content	16.723	1	16.723	115.73	<0.0001	**
A^2	17.110	1	17.110	118.50	<0.0001	**
B^2	47.336	1	47.336	327.75	<0.0001	**
C^2	17.654	1	17.654	122.18	<0.0001	**
D^2	12.184	1	12.184	84.36	<0.0001	**
A × B	0.925	1	0.925	6.40	0.0241	*
A × C	0.178	1	0.178	1.23	0.2857	n.s.
A × D	0.865	1	0.865	5.99	0.0284	*
B × C	0.003	1	0.003	0.02	0.8862	n.s.
B × D	0.011	1	0.011	0.08	0.7806	n.s.
C × D	0.925	1	0.925	6.40	0.0241	*
Residual	2.025	14	0.1446	–	–	–
Correlation total	203.367	28	–	–	–	–

Annotation: ** $p < 0.01$ (highly significant); * $p < 0.05$ (significant); n.s. = not significant.

Supplementary Table S4. Regression model summary statistics for UPE-Piperine.

Statistic	Value
Std. Dev.	0.412
Mean (Yield)	46.41
C.V. %	0.89
PRESS	10.744
R^2	0.9817
Adjusted R^2	0.9629
Predicted R^2	0.9012
Adeq Precision	19.52
